# Epidemiology and cardiometabolic care in adults with ASCVD and high 10-year ASCVD risk: 2021 WHO STEPS study in Iran

**DOI:** 10.1038/s41598-026-45344-0

**Published:** 2026-03-27

**Authors:** Hossein Farrokhpour, Maryam Nasserinejad, Naser Ahmadi, Ali Golestani, Mohammad Keykhaei, Sina Azadnajafabad, Arya Aminorroaya, Mohammadreza Naderian, Nazila Rezaei, Shirin Djalalinia, Yosef Farzi, Mohammad-Mahdi Rashidi, Negar Rezaei, Erfan Ghasemi, Elham Abdolhamidi, Moein Yoosefi, Ameneh Kazemi, Rosa Haghshenas, Mohamad Taghi Majnoon, Farshad Farzadfar

**Affiliations:** 1 Non-communicable Diseases Research Center, Endocrinology and Metabolism Population Sciences Institute, Tehran University of Medical Sciences, Tehran, Iran; 2 Development of Research and Technology Center, Deputy of Research and Technology, Ministry of Health and Medical Education, Tehran, Iran; 3 Endocrinology and Metabolism Research Center, Endocrinology and Metabolism Clinical Sciences Institute, Tehran University of Medical Sciences, Tehran, Iran; 4 Division of Pediatric Cardiology, Pediatrics Center of Excellence, School of Medicine, Children’s Medical Center, Tehran University of Medical Sciences, Tehran, Iran

**Keywords:** Atherosclerotic cardiovascular diseases, ASCVD, Epidemiology, Cardiometabolic risk factors, Iran, Cardiology, Diseases, Health care, Medical research, Risk factors

## Abstract

Atherosclerotic cardiovascular disease (ASCVD) remains the leading cause of mortality and health burden worldwide. We aimed to assess ASCVD epidemiology, 10-year risk, and cardiometabolic care using nationally representative data in Iran. Using the 2021 Iran STEPS study, a cross-sectional analysis was conducted to evaluate ASCVD (self-reported myocardial infarction, angina, revascularization, or stroke) prevalence and annual event rate. The 2013 AHA/ACC pooled cohort equations estimated 10-year ASCVD risk in 10,298 participants aged 40–75 without ASCVD. Cardiometabolic care for hypertension, diabetes mellitus (DM), and hypercholesterolemia was assessed, along with statin use and cardiovascular health (CVH) metrics. Among 27,822 participants, representing 57.5 million adults, ASCVD prevalence was 7.4%. Among those without ASCVD, 19.1% had an intermediate and 5.4% had a high ASCVD risk. In the ASCVD group, hypertension and DM affected 77.0% and 34.7%, yet only 12.5% and 14.3% of the entire ASCVD group had them controlled, respectively. In adults without ASCVD, 2.9–5.3% of the entire risk groups achieved hypertension targets, 2.5–16.8% achieved glycemic targets, and 7.7–16.7% achieved lipid goals, despite condition prevalence spanning 47.4–94.4% for hypertension, 7.2–59.3% for DM, and 33.5–48.7% for hypercholesterolemia. CVH metrics were also unfavorable. The study revealed a high ASCVD burden and future risks with gaps in coverage, treatment, and control.

**Supplementary Information:**

The online version contains supplementary material available at 10.1038/s41598-026-45344-0.

## Introduction

Cardiovascular diseases (CVDs) remain the number one threat to global health and the leading cause of mortality worldwide^1^. In 2022, 19.8 million deaths were attributed to CVDs, accounting for 396 million years of life lost. This was despite significant investment and health policy aimed at addressing the increasing trend in CVDs through various strategies and measures^2^. This is especially noteworthy given the preventable nature of modifiable CVD-related risk factors^3^.

The shift towards non-communicable diseases in Iran has received significant attention. Contributing factors include the aging population, the epidemiologic transition, and the consequent challenges in the health care system^4,5^. Atherosclerotic cardiovascular diseases (ASCVD) are at the forefront of this increase, with 40% of mortality attributed to CVD in Tehran in 2010^4,6^.

CVD has been the leading cause of mortality and disability-adjusted life years (DALYs) in Iran^7^. Iran is considered to have a high prevalence and incidence of ischemic heart diseases (IHDs) in the North Africa and Middle East (NAME) area^8^. Although the DALYs and death rates trends of IHDs in Iran have had more favorable results compared to other countries, demonstrating effective secondary and tertiary prevention^9,10^, they were still considerably higher than the global estimates^11^. These highlight the need for more comprehensive primary prevention measures and for addressing amenable risk factors. Besides, the current state of CVD and its future projections would play an essential role in helping health policymakers plan at a large scale and take measures to halt the rising disease burden.

Several ASCVD risk estimators have been developed using clinical data from various populations to evaluate the future chance of developing atherosclerotic CVDs^12–15^. Among them, the 2013 American Heart Association/American College of Cardiology (AHA/ACC) risk score has been validated in studies of the Iranian population^16,17^. However, it has not been used in any national-level study in Iran. While a previous report used data from 2016 studies to estimate future CVD status in Iran^18^, no study has used the validated 2013 AHA/ACC risk score with the most up-to-date data from the country. Additionally, using the 2013 AHA/ACC risk score allows for a valuable opportunity to evaluate the population’s behavioral, metabolic, and environmental factors on a national scale. This assessment enables clinicians to identify areas for improvement^19^.

Herein, using data from the nationally representative Iran STEPS survey 2021^20^, we set out to evaluate the prevalence of ASCVD in Iran at the national and subnational levels. We also aim to examine various factors associated with ASCVD and to provide a clear picture of the future risk of ASCVD in the non-clinically-presented population over the next 10 years. Additionally, we assess the status of various cardiometabolic health factors across different populations.

## Methods

### STEPS survey overview

This study was based on the data from the most recent nationally representative Iran STEPS Survey 2021, conducted according to the WHO STEPwise Approach to Non-Communicable Disease (NCD) Risk Factor Surveillance (STEPS) framework, whose protocol and details are provided elsewhere^20^. In short, the STEPS 2021 cross-sectional study comprised three steps: questionnaires, physical measurements, and laboratory assessments for data collection. In the first step, the latest version of the WHO STEPS instrument, version 3.2, was used and further modified to meet survey requirements and reflect the population characteristics. To avoid issues linked to paper questionnaires, an electronic questionnaire was used by the trained staff via tablets. In the physical measurement, height, weight, waist and hip circumference, blood pressure, and pulse rate were measured according to the predefined criteria in the protocol. Finally, the laboratory measurement was done with an auto-analyzer (Roche-Hitachi Cobas C311, High–Technologies Corporation, Tokyo, Japan) at the survey headquarters.

The study protocol adhered to the Declaration of Helsinki and was approved by the ethical committee of the National Institute for Health Research (ID: IR.TUMS.NIHR.REC.1398.006). All participants provided informed consent prior to participation in surveys.

### Inclusion criteria and study population

Iranians aged ≥ 18 years residing in rural and urban areas of all 31 provinces of Iran were recruited using a clustered sampling method. For the first, second, and third steps of the survey, 27,874, 27,745, and 18,119 individuals participated. Analyses on ASCVD were performed on individuals without missing values regarding ASCVD questions in the first step. Analyses of 10-year ASCVD risk were conducted on the 40–75 age group with complete data based on the third step of the STEPS survey.

### Variables and outcomes

The presence of ASCVD was determined based on participants’ reports, using a composite measure that included myocardial infarction, stable or unstable angina, coronary revascularization, and stroke. Subjects were asked, “Have you ever had or been told by medical staff that you’ve had a heart attack or chest pain from heart disease (angina)? Have you done cardiological interventions like angioplasty with a positive answer, stent or heart bypass surgery?”. A “yes” answer to any of those questions was considered a positive coronary artery disease. Patients were also asked if they were told by medical staff that they had a stroke (cerebrovascular accident or incident). A yes answer to that question was considered a positive history of stroke. A yes answer to any of those questions was considered as a positive history of ASCVD. To assess recent events, participants were asked the same set of questions with the phrase “during the last 12 months” to distinguish newly occurring cases from past events.

The recent annual event rate for ASCVD was calculated by defining the at-risk population as participants free of ASCVD at the start of the 12-month period, identified as those who answered ‘no’ to ever having ASCVD, combined with those who reported a new ASCVD event in the past 12 months. New cases were identified as participants answering ‘yes’ to experiencing an ASCVD event in the past 12 months. The recent annual event rate was then calculated as the number of new cases divided by the at-risk population, expressed as cases per 1000 participants per year, with 95% confidence intervals.

The revised 2013 AHA/ACC risk estimator was used to calculate the 10-year risk of ASCVD^13,21^. We used the revised version to compensate for the probable overestimation linked to the original pooled cohort equations (PCE)^21^. The calculation included age, race, sex, total cholesterol, HDL cholesterol, systolic blood pressure, presence of hypertension medications, diabetes mellitus (DM), and smoking status of the individuals.

Individuals with no current ASCVD were then categorized into two groups based on their age: age < 40 years, and 40 years ≤ age ≤ 75 years. The participants were further divided by the ASCVD risk score of < 5% as low, 5% ≤ ASCVD risk score < 7.5% as borderline, 7.5% ≤ ASCVD risk score < 20% as intermediate, and risk scores of ≥ 20% as high. The demographics, survey-derived, and laboratory characteristics, including lifestyle and metabolic risk factors, were evaluated in these groups.

DM was defined as taking anti-hyperglycemic drugs or having fasting plasma glucose (FPG) ≥ 126 mg/dL (7.0 mmol/L). DM awareness was determined among individuals with diabetes by self-reported history, obtained by asking whether a healthcare professional had ever informed them of having high blood sugar or diabetes. Treatment coverage for DM was assessed by asking participants if they were currently taking any oral antihyperglycemic medication or insulin. Effective DM was evaluated using whole blood Hemoglobin A1c (HbA1c) measurements, with good control defined as HbA1c < 7% and fair control as HbA1c < 8%^22^.

Hypertension was defined as systolic blood pressure (SBP) ≥ 140 mmHg or diastolic blood pressure (DBP) ≥ 90 mmHg or taking antihypertensive medications^23^. Blood pressure was measured three times for each participant by trained staff using calibrated standard sphygmomanometers. The first reading was excluded, and the average of the remaining two measurements was used for analysis. Awareness of hypertension was defined as a self-reported history of a healthcare professional diagnosis, consistent with the WHO definition and equivalent to previously described awareness measures. Treatment was defined as the proportion of individuals with hypertension who reported current use of antihypertensive medication. Control was defined as the proportion of treated individuals whose blood pressure was within the normal range according to each guideline.

Dyslipidemia was defined as the presence of one of the following: hypertriglyceridemia ≥ 150 mg/dL, LDL-C ≥ 130 mg/ dL, low HDL-C (< 50 mg/dL in women, < 40 mg/dL in men), and total cholesterol level of ≥ 200 mg/dL, or self-report of using lipid-lowering medications^24^. Hypercholesterolemia was defined as a total cholesterol level ≥ 200 mg/dL or self-report of lipid-lowering medication use^24^. Awareness was determined as individuals with hypercholesterolemia who reported having been previously diagnosed by a health professional. Treatment coverage referred to those aware of their condition who were receiving lipid-lowering medication. Effective control was defined as having a total cholesterol < 200 mg/dL.

The statin use among the eligible was evaluated in three populations: 1. All the population with reported ASCVD, 2. Participants with intermediate and high-risk 10-year ASCVD risk, 3. Individuals with DM and without ASCVD.

To assess cardiovascular health (CVH) metrics, this study employed the Life’s Simple 7 (LS7) framework. It encompasses seven key metabolic and behavioral risk factors, in line with the American Heart Association’s 2020 strategic goals for assessing ideal CVH^25^. These factors included current smoking status, body mass index (BMI, measured in kg/m^2^), physical activity levels, components of a healthy diet score, total cholesterol (mg/dL), blood pressure (mm Hg), and FPG (mg/dL), classified into three categories: poor, intermediate, and ideal. While six of the LS7 components aligned directly with the AHA’s established LS7 criteria, the healthy diet score was tailored to fit the STEPS survey’s measurement approach, with scoring adjusted to reflect the three-level criteria. Detailed information on the dietary questions, scoring approach, and classification of the healthy diet component is provided in Online Resource 1, Supplementary Method Information.

The socioeconomic status of the survey population was assessed using a wealth index (WI), derived from questionnaire data on household assets. The resulting WI values were divided into five quintiles, ranging from the poorest (first quintile) to the wealthiest (fifth quintile). Education level was evaluated based on years of schooling and classified into four groups: 0 years, 1–6 years, 7–11 years, and 12 or more years. Data on the other included variables can be accessed in the main protocol^20^.

### Statistical analysis

Two expert biostatisticians performed the data cleaning and weighting process. The most recent data on the Iranian population, extracted from the 2016 Iran Census. This data, representing 57.5 million Iranian adults, was used as the reference for standardizing age, sex, and area of residence^20^. Variables were presented as prevalence estimates (percentages per population) with 95% confidence intervals (95% CI). The prevalence of ASCVD and its estimation were also depicted in maps, divided by sex and age. The burden of ASCVD was evaluated by estimating its national prevalence. To generalize these findings to the national level, the sample prevalence was adjusted using age-, sex-, and urban/rural-residence-based weighting to align with the 2016 Iranian standard population. Statistical significance was defined as *p* < 0.05, or if the 95% CI between groups did not cross. All statistical analyses were performed using Stata version 14 (StataCorp, College Station, Texas, USA) and the R statistical package version 4.1.2 (https://cran.r-project.org).

## Results

### Overall prevalence, recent annual event rate, and characteristics of ASCVD

A total of 27,822 participants were included in the final analysis using the first step of the survey. Among these, 1840 subjects (6.7%, 95% CI: 6.3–7.0) were reported to have a history of coronary artery disease, and 395 subjects (1.4%, 95% CI: 1.2–1.5) had a history of stroke, comprising a total of 2046 participants (7.4%, 95% CI: 7.0%–7.7%) with ASCVD. The prevalence of ASCVD in participants aged 35 years or older was 10.9%. Additionally, 530 individuals reported experiencing an atherosclerotic cardiovascular event for the first time within the past year, corresponding to an estimated recent annual event rate of 20.1 (95% CI: 18.4–21.8) per 1000 people per year.

The baseline characteristics, demographics, and metabolic and lifestyle state of the total population are summarized in Table 1. In subjects with ASCVD and coronary artery disease, 96.3% and 96.5% were older than 35, respectively. Compared to participants with ASCVD, those without ASCVD exhibited significantly higher levels of education and wealth. (*p* < 0.001) Participants with ASCVD had higher hypertension, DM, dyslipidemia, and physical inactivity compared to subjects with no ASCVD. (*p* < 0.001) In terms of Doctors’ advocacy for weight loss, tobacco cessation, and reduced salt consumption in subjects with ASCVD compared to non-ASCVD participants, only the recommendation for lower salt consumption was higher in individuals with ASCVD.

**Table 1:** Baseline, dietary, and laboratory characteristics of the study population based on the presence of atherosclerotic cardiovascular disease.

Variable		Participants with cardiovascular disease			Participants with no cardiovascular disease	*p*-value*
Number		Total N = 2046 (7.4%)	Cardiac origin N = 1840 (6.7%)	Stroke N = 395 (1.4%)	N = 25,776 (92.5%)	
Age (years)		61.2 (60.59–61.81)	61.48 (60.85–62.11)	61.48 (60.02–62.95)	44.46 (44.25–44.66)	< 0.001
Sex (male)		1105 (54.4) (51.98,56.8)	1001 (54.91) (52.36,57.43)	215 (53.5) (47.95,58.97)	11,348 (43.84) (43.17,44.52)	< 0.001
BMI	Underweight (< 18.5)	34 (1.54%) (1.07–2.22)	32 (1.6%) (1.1–2.34)	4 (0.93%) (0.3–2.87)	933 (3.51%) (3.28–3.76)	< 0.001
	Normal (18.5 ≤ < 25)	515 (24.32%) (22.32–26.45)	451 (23.76%) (21.67–25.98)	119 (29.21%) (24.44–34.49)	8904 (34.37%) (33.72–35.02)	
	Overweight (25 ≤ < 30)	842 (41.14%) (38.77–43.55)	766 (41.68%) (39.18–44.22)	153 (37.68%) (32.46–43.2)	9626 (37.79%) (37.12–38.45)	
	Obesity (30 ≤ BMI)	647 (33%) (30.75–35.33)	586 (32.96%) (30.59–35.41)	115 (32.17%) (27.16–37.63)	6183 (24.33%) (23.75–24.93)	
SBP (mmHg)		136.19 (135.19–137.2)	135.79 (134.75–136.84)	139.01 (136.5–141.52)	125.03 (124.78–125.27)	< 0.001
DBP (mmHg)		80.16 (79.57–80.75)	79.91 (79.3–80.52)	80.81 (79.22–82.41)	77.59 (77.44–77.74)	< 0.001
Triglycerides (mg/dL)		156 (149.44–162.56)	156.36 (149.4–163.32)	151.54 (149.67–153.4)	151.18 (149.26–153.11)	0.16
LDL-C (mg/dL)		87.07 (84.52–89.62)	86.3 (83.65–88.96)	91.41 (85.79–97.03)	100.61 (99.89–101.33)	< 0.001
HDL-C (mg/dL)		40.51 (39.79–41.24)	40.51 (39.79–41.24)	41.27 (39.72–42.81)	42.38 (42.16–42.6)	< 0.001
*Social status:*						
Marital status (married)	Single	58 (3.03%) (2.29–4.01)	47 (2.72%) (1.99–3.72)	13 (3.79%) (2.13–6.67)	4173 (15.97%) (15.48–16.47)	< 0.001
	Married	1658 (80.65%) (78.66–82.49)	1494 (80.77%) (78.68–82.7)	320 (79.58%) (74.71–83.72)	19,667 (76.3%) (75.72–76.87)	
	Divorced	31 (1.65%) (1.13–2.4)	30 (1.75%) (1.19–2.56)	5 (1.68%) (0.69–4.05)	534 (2.24%) (2.04–2.45)	
	Widowed	299 (14.67%) (13.04–16.46)	269 (14.76%) (13.05–16.65)	57 (14.95%) (11.4–19.35)	1402 (5.5%) (5.2–5.82)	
Years of education	0 (illiterate)	622 (28.02%) (25.93–30.2)	558 (28.01%) (25.82–30.31)	124 (28.72%) (24.09–33.84)	3385 (12.3%) (11.87–12.74)	< 0.001
	1–7 years(primary)	675 (32.65%) (30.42–34.95)	602 (32.3%) (29.97–34.72)	144 (36.66%) (31.5–42.15)	6064 (23.1%) (22.53–23.67)	< 0.001
	7–12 years	256 (13.82%) (12.19–15.63)	228 (13.85%) (12.13–15.76)	44 (11.62%) (8.44–15.78)	4928 (19.52%) (18.99–20.07)	< 0.001
	12 + years(academic)	477 (25.52%) (23.42–27.74)	438 (25.84%) (23.63–28.19)	80 (23%) (18.54–28.17)	11,216 (45.08%) (44.41–45.75)	< 0.001
Wealth index	1	478 (22.08%) (20.16–24.13)	423 (21.86%) (19.84–24.01)	95 (22.16%) (17.97–27.01)	4786 (18.78%) (18.26–19.32)	< 0.001
	2	440 (23.23%) (21.2–25.39)	385 (22.52%) (20.42–24.78)	98 (27.27%) (22.51–32.61)	4820 (20.43%) (19.87–21)	< 0.001
	3	393 (19.32%) (17.46–21.33)	344 (18.82%) (16.89–20.92)	86 (21.8%) (17.53–26.77)	4865 (18.9%) (18.37–19.44)	< 0.001
	4	369 (18.56%) (16.73–20.54)	348 (19.35%) (17.39–21.47)	56 (15.06%) (11.47–19.54)	4893 (20.1%) (19.54–20.66)	< 0.001
	5	302 (16.81%) (15–18.78)	283 (17.45%) (15.52–19.55)	51 (13.71%) (10.27–18.06)	4955 (21.79%) (21.22–22.38)	< 0.001
Salt consumption self-awareness	Average or less	1818 (89%) (87.39–90.42)	1631 (88.85%) (87.15–90.35)	351 (88.78%) (84.76–91.84)	22,301 (87.19%) (86.74–87.63)	0.025
	Much or higher	228 (11%) (9.58–12.61)	209 (11.15%) (9.65–12.85)	44 (11.22%) (8.16–15.24)	3446 (12.81%) (12.37–13.26)	
Alcohol consumption history (ever)		152 (8.48%) (7.19–9.99)	144 (8.94%) (7.55–10.57)	19 (5.57%) (3.44–8.89)	1616 (6.73%) (6.39–7.08)	0.017
Alcohol consumption (During the past 30 days, did you have at least six or more standard drinks in a single drinking occasion?)		9 (0.49%) (0.24–1)	9 (0.54%) (0.26–1.11)	0	219 (0.94%) (0.81–1.09)	0.019
Hypertension		1573 (77.05%) (74.96–79.02)	1413 (77.08%) (74.88–79.14)	313 (77.97%) (72.9–82.32)	11,857 (45.06%) (44.38–45.74)	< 0.001
Hypertension awareness		1170 (56.84%) (54.42–59.23)	1052 (56.98%) (54.43–59.48)	245 (61.16%) (55.59–66.46)	4380 (16.7%) (16.2–17.21)	< 0.001
Hypertension coverage		1090 (52.96%) (50.53–55.37)	979 (53.05%) (50.5–55.59)	231 (58.1%) (52.51–63.49)	3582 (13.74%) (13.27–14.21)	< 0.001
Hypertension effective care	SBP < 140 and DBP < 90	473 (23.08%) (21.1–25.19)	431 (23.44%) (21.34–25.67)	88 (22.03%) (17.83–26.9)	1441 (5.68%) (5.37–6.01)	< 0.001
	SBP < 130 (mmHg) and DBP < 80 (mmHg)	249 (12.53%) (10.99–14.24)	227 (12.85%) (11.22–14.68)	46 (10.98%) (8.03–14.83)	565 (2.29%) (2.1–2.51)	
Diabetes mellitus		493 (34.79%) (31.44–38.29)	449 (35.52%) (31.97–39.23)	91 (32.61%) (25.41–40.74)	1946 (12.25%) (11.55–12.98)	< 0.001
Diabetes mellitus awareness		407 (29.2%) (26.01–32.6)	368 (29.68%) (26.31–33.29)	82 (29.55%) (22.53–37.68)	1394 (8.62%) (8.03–9.24)	< 0.001
Diabetes mellitus coverage		373 (26.43%) (23.38–29.73)	337 (26.88%) (23.65–30.38)	76 (27.11%) (20.3–35.21)	1220 (7.6%) (7.04–8.19)	< 0.001
Diabetes mellitus effective care	HbA1c < 7%	99 (7.39%) (5.7–9.53)	90 (7.3%) (5.55–9.54)	20 (12.96%) (7.45–21.58)	350 (2.06%) (1.79–2.37)	< 0.001
	HbA1c < 8%	200 (14.34%) (11.86–17.23)	184 (14.59%) (11.97–17.66)	37 (16.69%) (10.76–24.98)	597 (3.72%) (3.34–4.14)	
Hypercholesterolemia		735 (48.3%) (44.74–51.87)	674 (49.06%) (45.3–52.82)	141 (49.57%) (41.66–57.5)	4799 (28.91%) (27.93–29.91)	
Hypercholesterolemia awareness		583 (38.28%) (34.88–41.79)	536 (39.42%) (35.81–43.14)	109 (34.13%) (27.29–41.71)	2256 (13.24%) (12.56–13.95)	
Hypercholesterolemia coverage		526 (35.05%) (31.71–38.53)	489 (36.45%) (32.9–40.16)	96 (30.63%) (24.07–38.09)	1555 (9.16%) (8.6–9.75)	
Hypercholesterolemia effective care (total cholesterol < 200 mg/dL)		461 (30.85%) (27.63–34.26)	427 (32.04%) (28.6–35.68)	84 (27.24%) (20.97–34.55)	1242 (7.35%) (6.84–7.89)	
Dyslipidemia definition		1026 (69.62%) (66.31–72.73)	933 (69.68%) (66.17–72.97)	188 (70.4%) (62.96–76.9)	10,173 (61.22%) (60.16–62.28)	< 0.001
Current smoking		282 (14.16%) (12.54–15.94)	260 (14.64%) (12.91–16.55)	52 (13.4%) (10.04–17.67)	3516 (13.99%) (13.52–14.47)	< 0.001
Physical inactivity (< 600 METs)		1112 (58.5%) (56.04–60.93)	988 (57.83%) (55.23–60.38)	245 (68.52%) (62.99–73.57)	11,681 (50.72%) (50.01–51.43)	< 0.001
Statin therapy		1158 (56.49%) (54.08–58.88)	1088 (59.08%) (56.55–61.56)	198 (49.66%) (44.15–55.18)	1618 (6.38%) (6.05–6.72)	< 0.001
Aspirin intake		1423 (70%) (67.74–72.16)	1308 (71.54%) (69.2–73.78)	267 (67.98%) (62.59–72.93)	2810 (10.92%) (10.5–11.35)	< 0.001
Family history of heart attack, stroke or sudden death		379 (20.23%) (18.31–22.31)	345 (20.6%) (18.56–22.8)	77 (21.04%) (16.77–26.04)	2852 (11.79%) (11.34–12.25)	< 0.001
Doctors’ recommendation for tobacco cessation in the past 12 months		1001 (48.69%) (46.27–51.12)	901 (48.74%) (46.19–51.3)	200 (50.88%) (45.33–56.41)	12,488 (48.02%) (47.35–48.7)	0.605
Doctors’ recommendation for lowering salt consumption in the past 12 months		1546 (76.22%) (74.09–78.22)	1397 (76.5%) (74.27–78.59)	295 (74.4%) (69.17–79.01)	18,164 (70.58%) (69.95–71.2)	< 0.001
Doctors’ recommendation for doing exercise in the past 12 months		1481 (72.52%) (70.3–74.64)	1334 (72.45%) (70.11–74.68)	282 (72.41%) (67.21–77.07)	18,582 (72.27%) (71.66–72.88)	0.826
Doctors’ recommendation for lowering weight in the past 12 months		1444 (71.1%) (68.86–73.25)	1302 (71.14%) (68.77–73.4)	267 (68.31%) (62.91–73.25)	17,782 (68.92%) (68.28–69.55)	0.062

Data are presented as mean (confidence interval) or for continuous variables and n (% and confidence interval) for categorical variables as appropriate.

*p-value between patients with total cardiovascular diseases and the population without self-reported atherosclerotic cardiovascular disease group.

ASCVD, Atherosclerotic cardiovascular disease; BMI, Body mass index; DBP, diastolic blood pressure; HDL, High-density lipoprotein; LDL, Low-density lipoprotein cholesterol; METs, Metabolic equivalent of task; SBP, Systolic blood pressure.

Based on the overall data, approximately 1.73 million females and 2.59 million males, totaling 4.32 million individuals, were affected by ASCVD at the national level, with the majority being male (61.2%) and residing in urban areas (77.3%). (Table 2).

**Table 2:** National prevalence of atherosclerotic cardiovascular diseases in Iranian adults aged ≥ 18 years.

Sex	Area	Cardiac disease	Stroke	Cardiovascular diseases
		Prevalence (%, 95% CI)	Prevalence (%, 95% CI)	Prevalence (%, 95% CI)
Male	Rural	491,950.5 (419,483.8–564,417.3)	119,441.6 (83,525.2–155,358)	560,147.4 (483,416.3–636,878.6)
	Urban	1,874,453.5 (1,735,849.6–2,013,057.5)	362,098.2 (300,163.2–424,033.2)	2,033,085.7 (1,889,684.8–2,176,486.6)
	All areas	2,370,202.5 (2,213,585.6–2,526,819.4)	481,696.1 (410,087.4–553,304.8)	2,596,724.5 (2,433,929.1–2,759,519.9)
Female	Rural	351,125.1 (297,926.4–404,323.9)	84,999.8 (57,895.6–112,103.9)	411,984.5 (354,426.3–469,542.7)
	Urban	1,196,355 (1,096,125.1–1,296,585)	247,926.7 (200,851.7–295,001.7)	1,318,871.2 (1,213,929.2–1,423,813.3)
	All areas	1,547,955.7 (1,434,429.9–1,661,481.5)	332,907 (278,581.8–387,232.2)	1,731,139.7 (1,611,410.4–1,850,869)
Total	Rural	828,164.8 (740,621.5–915,708)	200,790.1 (156,860.2–244,720)	956,664.5 (863,028.7–1,050,300.2)
	Urban	2,995,712.2 (2,829,300.4–3,162,124.1)	597,398.5 (521,437.7–673,359.3)	3,272,883.1 (3,099,773.8–3,445,992.4)
	All areas	3,827,310.8 (3,639,185.7–4,015,436)	798,244.4 (710,485.6–886,003.2)	4,232,383.6 (4,035,529.7–4,429,237.6)

Data are presented as numbers (95% confidence interval).

### Provincial ASCVD prevalence and recent annual event rate

**Fig. 1:**
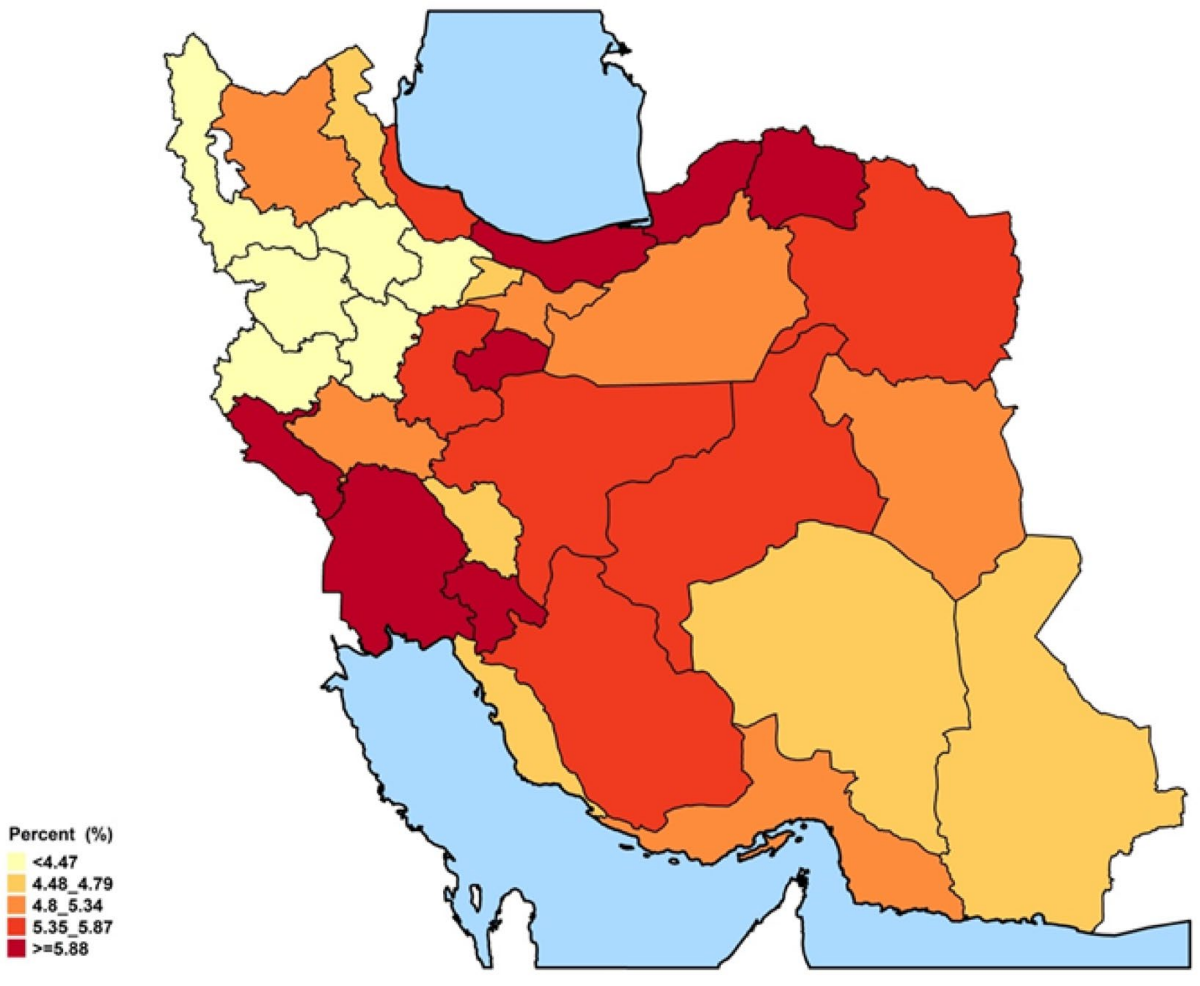
Subnational and provincial prevalence of total ASCVD ASCVD: atherosclerotic cardiovascular disease. The figure was generated using ggplot2 from the R statistical package version 4.1.2 (https://cran.r-project.org).

**Fig. 2:**
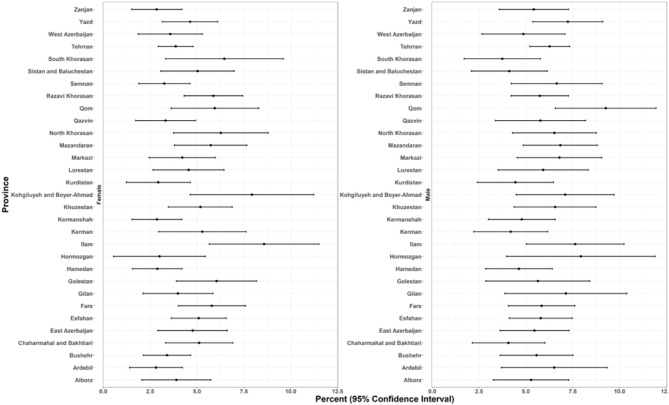
Gender and geographical distribution of ASCVD prevalence. ASCVD: atherosclerotic cardiovascular disease.

**Fig. 3:**
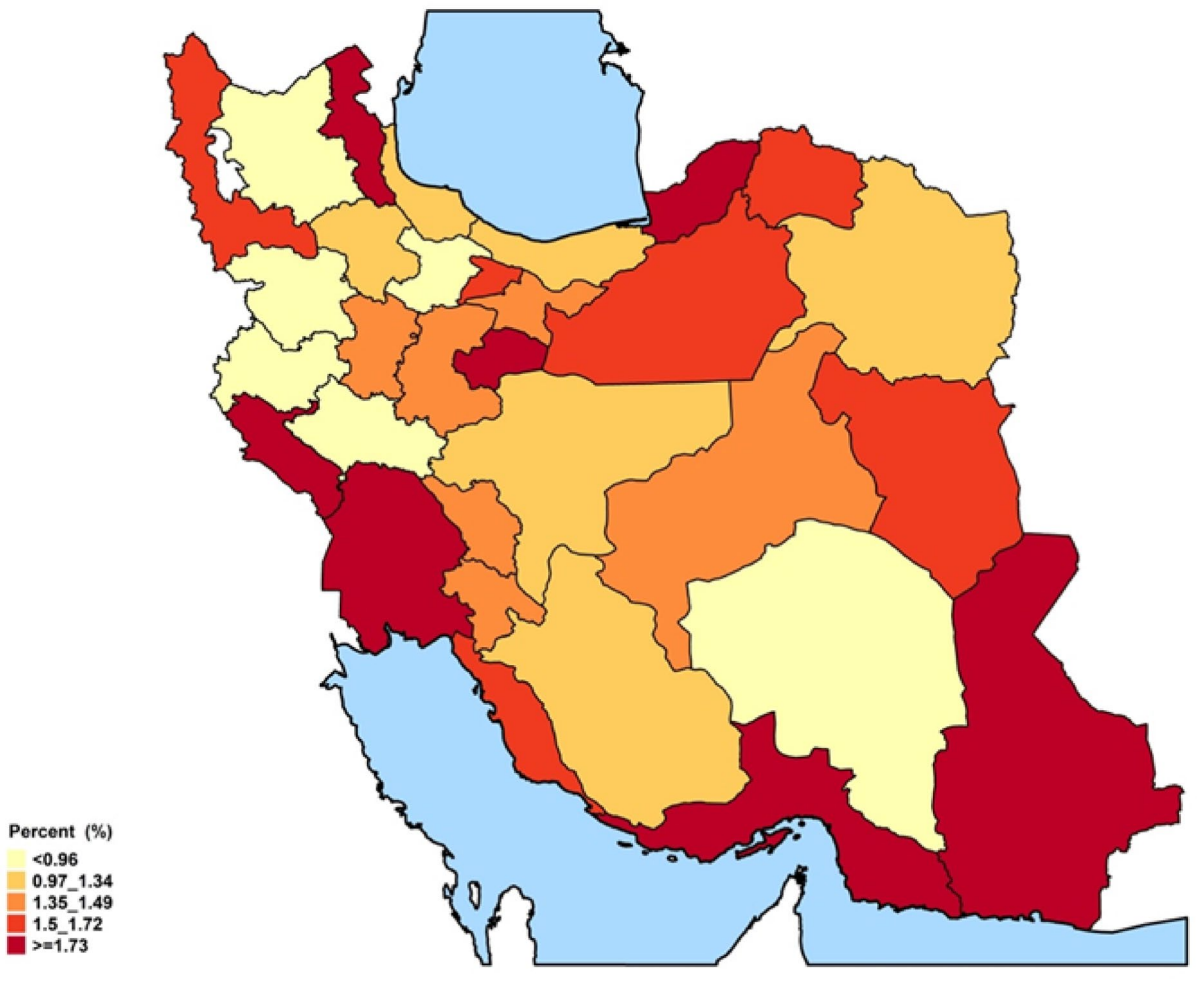
Subnational and provincial recent annual event rate of ASCVD ASCVD: atherosclerotic cardiovascular disease. The figure was generated using ggplot2 from the R statistical package version 4.1.2 (https://cran.r-project.org).

**Fig. 4:**
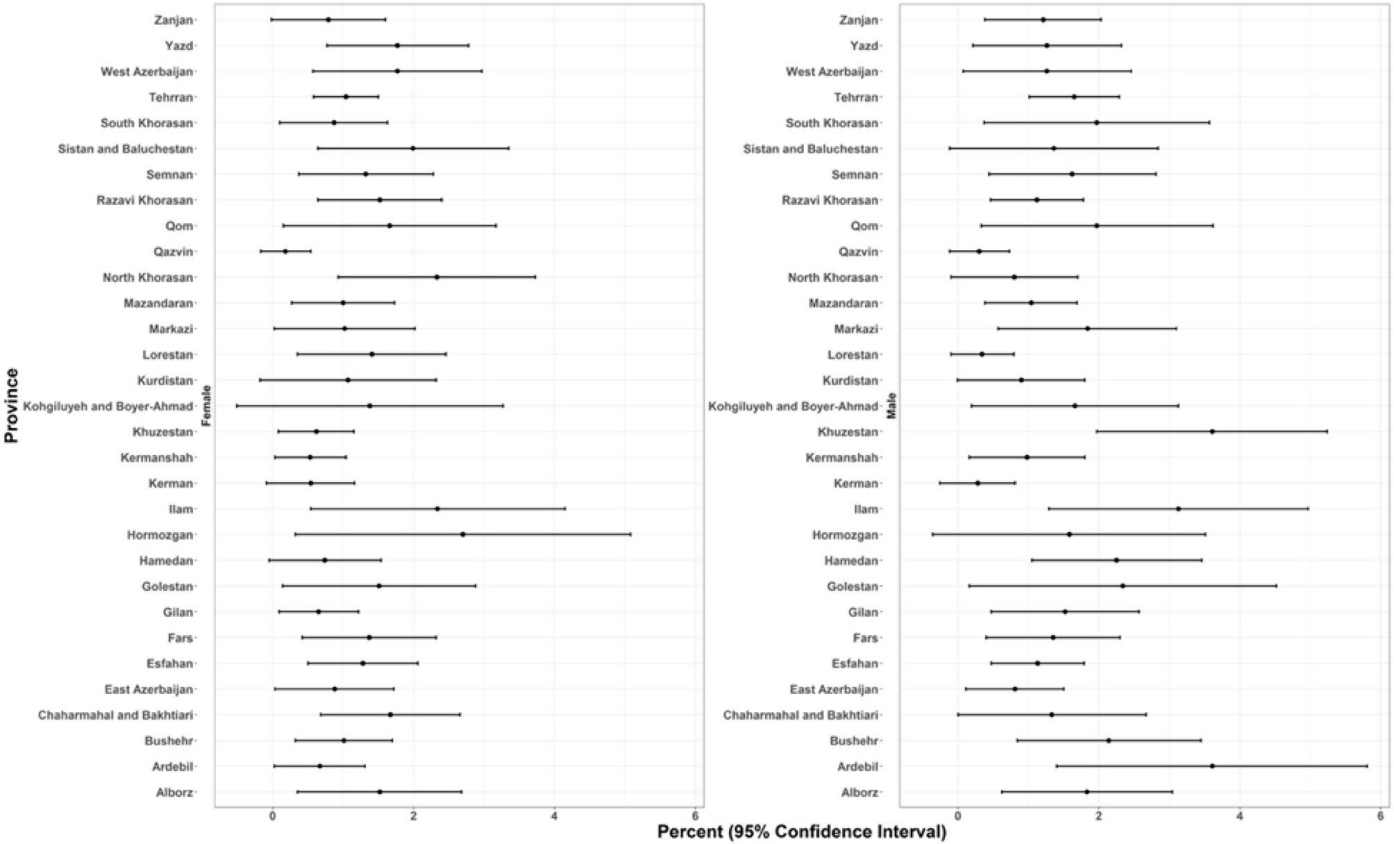
Gender and geographical distribution of ASCVD recent annual event rate. ASCVD: atherosclerotic cardiovascular disease.

The spatial distribution revealed some distinct geographical patterns. Provinces in the northeast and southwest regions, encompassing both coastal and inland areas, exhibited the highest overall prevalence of ASCVD (Figs. 1, 2, and Online Resource 1, Supplementary Fig. S1). In males, prevalence peaked in the central, western, and southern coastal areas. In females, prevalence was elevated in the southwestern, western, and eastern regions. At the provincial level, ASCVD prevalence ranged from approximately 2.6% to 8.6% in females and from 3.7% to 9.2% in males (Fig. 2). Stroke prevalence was notably higher in males. While the prevalence of stroke in men was higher along the northern and southern coastal lines, females had higher rates in western and southern inland provinces (Online Resource 1, Supplementary Fig. S2). Regarding the ASCVD recent annual event rate, provinces in the southeast, south, and west of the country were found to have high rates. Notably, the recent annual event rate was less pronounced in the central parts. (Figs. 3,4, and Online Resource 1, Supplementary Fig. S3 and S4). Ilam consistently appeared among the top-ranked provinces in both prevalence and recent annual event rate, emerging as a persistent regional hotspot.

### Estimated 10-year ASCVD risk in participants without prior ASCVD

The 2013 AHA/ACC ASCVD risk score was estimated in the 10,298 study subjects with available required data aged 40–75 without a history of ASCVD. While 64.3% (CI: 63.3–65.3) had low risk (defined as ASCVD risk score of < 5%), 11.1% (CI: 10.4–11.8) had borderline risk (defined as 5% ≤ ASCVD risk score < 7.5%), 19.1% (CI: 18.2–19.9) had intermediate (defined as 7.5% ≤ ASCVD risk score < 20%), and 5.4% (CI: 4.9–5.9) had high-risk (defined as ASCVD risk score ≥ 20%). The demographic and other characteristics of this population are presented in Table 3. Men had higher ASCVD risk scores compared to women.

**Table 3:** Baseline, dietary, and laboratory characteristics in participants with no report of atherosclerotic cardiovascular disease.

Variable		Age				
		20–39 years (N = 11,002)	40–75 years (N = 10,298)			
			ASCVD < 5% N = 6624 (64.34%) (63.3–65.36)	5% ≤ ASCVD < 7.5% N = 1154 (11.15%) (10.49–11.84)	7.5% ≤ ASCVD < 20% N = 1942 (19.1%) (18.26–19.97)	20% ≤ ASCVD N = 578 (5.42%) (4.95–5.92)
Age (year)		34.57 (33.95–35.19)	49.64 (49.08–50.2)	55.23 (54.37–56.09)	59.96 (59.35–60.56)	65.28 (64.47–6.09)
Sex (male)		4874 (44.29%) (43.26–45.33)	1969 (29.86%) (28.39–31.39)	687 (59.07%) (55.14–62.89)	1286 (67.08%) (63.64–70.35)	434 (73.61%) (68.49–78.16)
*Social status*:						
Wealth index	1	1860 (17.48%) (16.69–18.29)	1183 (17.24%) (16.05–18.49)	235 (17.88%) (15.19–20.93)	456 (20.73%) (17.84–23.95)	165 (26.32%) (21.4–31.92)
	2	1995 (19.86%) (19.01–20.74)	1134 (19.55%) (18.17–21.01)	217 (20.29%) (17.25–23.7)	368 (19.99%) (17.41–22.85)	133 (23.29%) (19.1–28.07)
	3	2127 (19.58%) (18.74–20.44)	1348 (19.06%) (17.89–20.29)	234 (18.07%) (15.51–20.94)	384 (18.78%) (16.45–21.37)	102 (17.74%) (14.17–21.98)
	4	2158 (20.84%) (19.98–21.73)	1277 (20.63%) (19.29–22.03)	232 (20.22%) (17.34–23.44)	364 (19.73%) (17.34–22.36)	97 (18.07%) (14.07–22.91)
	5	2113 (22.24%) (21.34–23.16)	1258 (23.53%) (21.97–25.15)	205 (23.55%) (19.88–27.65)	314 (20.77%) (17.59–24.35)	67 (14.58%) (10.72–19.52)
Marital status:	Single	3727 (33.8%) (32.82–34.79)	275 (4.4%) (3.73–5.19)	18 (1.56%) (0.89–2.72)	16 (1.14%) (0.57–2.27)	2 (0.31%) (0.08–1.26)
	Married	6983 (63.46%) (62.45–64.46)	5811 (87.03%) (85.81–88.15)	1006 (87.13%) (84.47–89.39)	1687 (86.64%) (84.17–88.77)	498 (84.28%) (78.7–88.61)
	Divorced	237 (2.24%) (1.95–2.57)	150 (2.84%) (2.23–3.6)	23 (1.84%) (1.12–3)	28 (1.5%) (0.97–2.31)	10 (4.05%) (1.34–11.6)
	widowed	55 (0.51%) (0.38–0.68)	388 (5.74%) (5.05–6.51)	107 (9.47%) (7.51–11.87)	211 (10.72%) (8.78–13.01)	68 (11.37%) (8.65–14.79)
Years of education:	0 (illiterate)	195 (1.61%) (1.38–1.89)	1086 (13.84%) (12.86–14.88)	273 (18.79%) (16.28–21.58)	612 (24.15%) (21.89–26.58)	226 (34.16%) (29.52–39.12)
	1–6 years(primary)	1361 (11.86%) (11.21–12.53)	2280 (30.97%) (29.55–32.43)	380 (33.66%) (29.99–37.53)	623 (32.2%) (28.99–35.6)	190 (35.22%) (30.17–40.64)
	7–12 years	2462 (22.23%) (21.38–23.11)	1234 (20.69%) (19.34–22.1)	189 (17.64%) (14.86–20.82)	271 (15.53%) (13.32–18.03)	58 (12.44%) (8.99–16.96)
	12 + (academic)	6897 (64.3%) (63.3–65.28)	1986 (34.51%) (32.85–36.21)	301 (29.92%) (26.19–33.92)	429 (28.11%) (24.64–31.87)	99 (18.18%) (13.55–23.95)
Salt consumption self-awareness	Average or less	9306 (85.15%) (84.4–85.86)	5791 (87.81%) (86.68–88.86)	1019 (87.86%) (84.82–90.36)	1722 (89.42%) (87.43–91.13)	515 (89.66%) (85.38–92.79)
	Much or higher	1685 (14.85%) (14.14–15.6)	830 (12.19%) (11.14–13.32)	135 (12.14%) (9.64–15.18)	218 (10.58%) (8.87–12.57)	62 (10.34%) (7.21–14.62)
Alcohol consumption (During the past 30 days, did you have at least six or more standard drinks in a single drinking occasion?)		171 (78.94) (72.45–84.23)	17 (47.53) (30–65.7)	5 (19.14) (7.82–9.76)	9 (25.22) (12.56–44.19)	2 (8.11) (2.05–27.13)
BMI	Underweight (< 18.5)	612 (5.53%) (5.08–6.02)	139 (1.75%) (1.41–2.17)	23 (1.43%) (0.9–2.26)	42 (1.85%) (1.17–2.89)	14 (1.76%) (0.99–3.12)
	Normal (18.5 ≤ < 25)	4860 (44.18%) (43.15–45.23)	1681 (25.07%) (23.64–26.55)	326 (29.42%) (25.81–33.32)	589 (28.75%) (25.81–31.88)	166 (27.94%) (23.21–33.22)
	Overweight (25 ≤ < 30)	3652 (33.66%) (32.67–34.66)	2650 (41.51%) (39.87–43.18)	471 (41.77%) (37.9–45.74)	779 (39.89%) (36.56–43.31)	248 (43.52%) (38.05–49.16)
	Obesity (30 ≤ BMI)	1814 (16.63%) (15.86–17.42)	2148 (31.67%) (30.19–33.19)	333 (27.38%) (24.18–30.82)	529 (29.52%) (26.21–33.05)	150 (26.78%) (22.29–31.8)
SBP (mmHg)		123.4 (121.19–125.61)	124.63 (123.31–125.94)	131.98 (130–133.95)	139.53 (137.97–141.08)	153.94 (151.48–156.4)
DBP (mmHg)		78.62 (77.04–80.2)	77.97 (77.01–78.92)	80.28 (78.76–81.8)	82.15 (81.15–83.15)	87.05 (85.57–88.53)
Hypertension		2808 (24.46%) (23.58–25.36)	3359 (47.4%) (45.75–49.05)	870 (73.27%) (69.4–76.81)	1606 (80.5%) (77.43–83.24)	547 (94.41%) (91.44–96.39)
Hypertension awareness		313 (2.7%) (2.39–3.06)	1168 (16.77%) (15.58–18.03)	425 (36.32%) (32.6–40.21)	875 (41.76%) (38.56–45.04)	315 (54.81%) (49.29–60.23)
Hypertension coverage		145 (1.3%) (1.09–1.57)	899 (13.11%) (12.05–14.24)	352 (31.29%) (27.69–35.12)	756 (36.29%) (33.29–39.4)	282 (47.29%) (41.82–52.82)
Hypertension effective care	SBP < 140 mmHg and DBP < 90 mmHg	77 (0.72%) (0.56–0.92)	464 (7.1%) (6.28–8.02)	140 (13.42%) (11–16.26)	217 (11.37%) (9.59–13.43)	70 (12.07%) (8.82–16.29)
	SBP < 130 mmHg and DBP < 80 mmHg	38 (0.36%) (0.25,0.52)	190 (2.96%) (2.47–3.54)	57 (5.38%) (3.88–7.42)	89 (4.76%) (3.63–6.2)	20 (4.17%) (2.06–8.26)
Diabetes mellitus		427 (3.8%) (3.0–4.6)	482 (7.28%) (6.68–7.93)	236 (20.45%) (18.22–22.87)	610 (31.41%) (29.38–33.51)	343 (59.34%) (55.29–63.27)
Diabetes mellitus awareness		75 (1.38%) (0.98–1.93)	349 (5.5%) (4.78–6.33)	174 (15.7%) (13.03–18.79)	471 (24.45%) (21.74–27.39)	248 (44.27%) (38.84–49.84)
Diabetes mellitus coverage		61 (1.1%) (0.75–1.62)	297 (4.8%) (4.11–5.61)	150 (14.24%) (11.64–17.3)	431 (21.84%) (19.32–24.59)	213 (38.46%) (33.18–44.03)
Diabetes mellitus effective care	HbA1c < 7%	23 (0.38%) (0.24–0.61)	92 (1.26%) (0.97–1.64)	51 (5.1%) (3.64–7.11)	105 (5.02%) (3.91–6.43)	63 (10.84%) (7.8–14.89)
	HbA1c < 8%	32 (0.53%) (0.36–0.79)	152 (2.52%) (2.03–3.12)	82 (8.02%) (6.12–10.45)	203 (9.79%) (8.22–11.62)	100 (16.89%) (13.19–21.36)
Hypercholesterolemia		772 (13.49%) (12.29–14.79)	2204 (33.52%) (31.97–35.11)	470 (39.15%) (35.36–43.07)	852 (45.28%) (41.78–48.82)	285 (48.74%) (43.22–54.28)
Hypercholesterolemia awareness		214 (3.58%) (2.99–4.28)	1025 (15.22%) (14.11–16.41)	246 (20.78%) (17.77–24.15)	490 (24.89%) (22.21–27.78)	167 (27.57%) (23.06–32.59)
Hypercholesterolemia coverage		89 (1.72%) (1.32–2.24)	675 (9.96%) (9.04–10.96)	185 (15.07%) (12.7–17.78)	382 (19.2%) (16.97–21.65)	135 (22.6%) (18.4–27.44)
Hypercholesterolemia effective care (Total cholesterol < 200 mg/dL)		69 (1.37%) (1–1.86)	532 (7.78%) (6.96–8.68)	151 (12.12%) (9.98–14.64)	315 (15.98%) (13.91–18.29)	98 (16.74%) (13.06–21.21)
Dyslipidemia		3296 (57.06%) (55.62–58.49)	3867 (57.79%) (56.12–59.44)	806 (67.6%) (63.71–71.27)	1397 (73.5%) (70.7–76.12)	481 (84.59%) (80.6–87.88)
Current Smoking		1455 (14.14%) (13.41–14.89)	425 (6.22%) (5.5–7.03)	221 (18.38%) (15.52–21.64)	527 (26.47%) (23.73–29.42)	255 (43.66%) (38.12–49.37)
Physical inactivity (< 600 METs) (%)		4709 (48.67%) (47.56–49.77)	2947 (51.57%) (49.83–53.3)	481 (48.62%) (44.43–52.82)	851 (49.78%) (46.07–53.49)	268 (53.93%) (48.24–59.51)
Triglycerides (mg/dL)		241.83 (193.35–290.32)	177.5 (162.24–192.76)	186.7 (172.08–201.31)	187.7 (178.11–197.29)	211.2 (194.51–227.89)
LDL-C (mg/dL)		94.43 (85.62–103.24)	90.81 (86.38–95.25)	90.44 (83.03–97.85)	90.83 (87.62–94.04)	98.2 (93.13–103.26)
HDL-C (mg/dL)		37.86 (36.2–39.52)	43.06 (41.94–44.18)	41.02 (39.44–42.61)	40.55 (39.38–41.71)	36.95 (35.71–38.19)
Self-reported medication for HTN		145 (1.3%) (1.08–1.56)	899 (13.11%) (12.05–14.24)	352 (31.29%) (27.69–35.12)	756 (36.29%) (33.29–39.4)	282 (47.29%) (41.82–52.82)
Self-reported medication for DM		97 (0.88%) (0.7–1.1)	297 (4.81%) (4.11–5.61)	150 (14.24%) (11.64–17.3)	431 (21.84%) (19.32–24.59)	213 (38.46%) (33.18–44.03)
Statin therapy		104 (0.96%) (0.77–1.19)	493 (7.49%) (6.65–8.42)	141 (13.22%) (10.55–16.44)	307 (14.4%) (12.56–16.45)	116 (18.92%) (15.08–23.47)
Aspirin therapy		223 (2.03%) (1.76–2.35)	711 (9.92%) (9.07–10.83)	263 (23.54%) (20.3–27.11)	597 (31.43%) (28.19–34.87)	212 (39.39%) (33.87–45.18)
Doctors’ recommendation for tobacco cessation in the past 12 months		5331 (48.48%) (47.43–49.52)	3137 (46.27%) (44.62–47.93)	583 (49.56%) (45.61–53.52)	942 (49.18%) (45.72–52.64)	306 (55.06%) (49.57–60.44)
Doctors’ recommendation for lowering salt consumption in the past 12 months		7404 (67.36%) (66.37–68.34)	4847 (72.88%) (71.32–74.37)	866 (77.4%) (74.18–80.33)	1416 (74.26%) (71.32–77)	422 (72.53%) (67.49–77.05)
Doctors’ recommendation to do exercise in the past 12 months		7898 (72.29%) (71.34–73.21)	4902 (73.35%) (71.8–74.85)	862 (77.77%) (74.57–80.68)	1348 (70.55%) (67.46–73.46)	405 (70.21%) (64.93–75.01)

Data are presented as mean (confidence interval) or for continuous variables and n (% and confidence interval) for categorical variables as appropriate.

ASCVD, Atherosclerotic cardiovascular disease; BMI, Body mass index; HDL, High-density lipoprotein; LDL, Low-density lipoprotein cholesterol; METs, Metabolic equivalent of task.

**Fig. 5:**
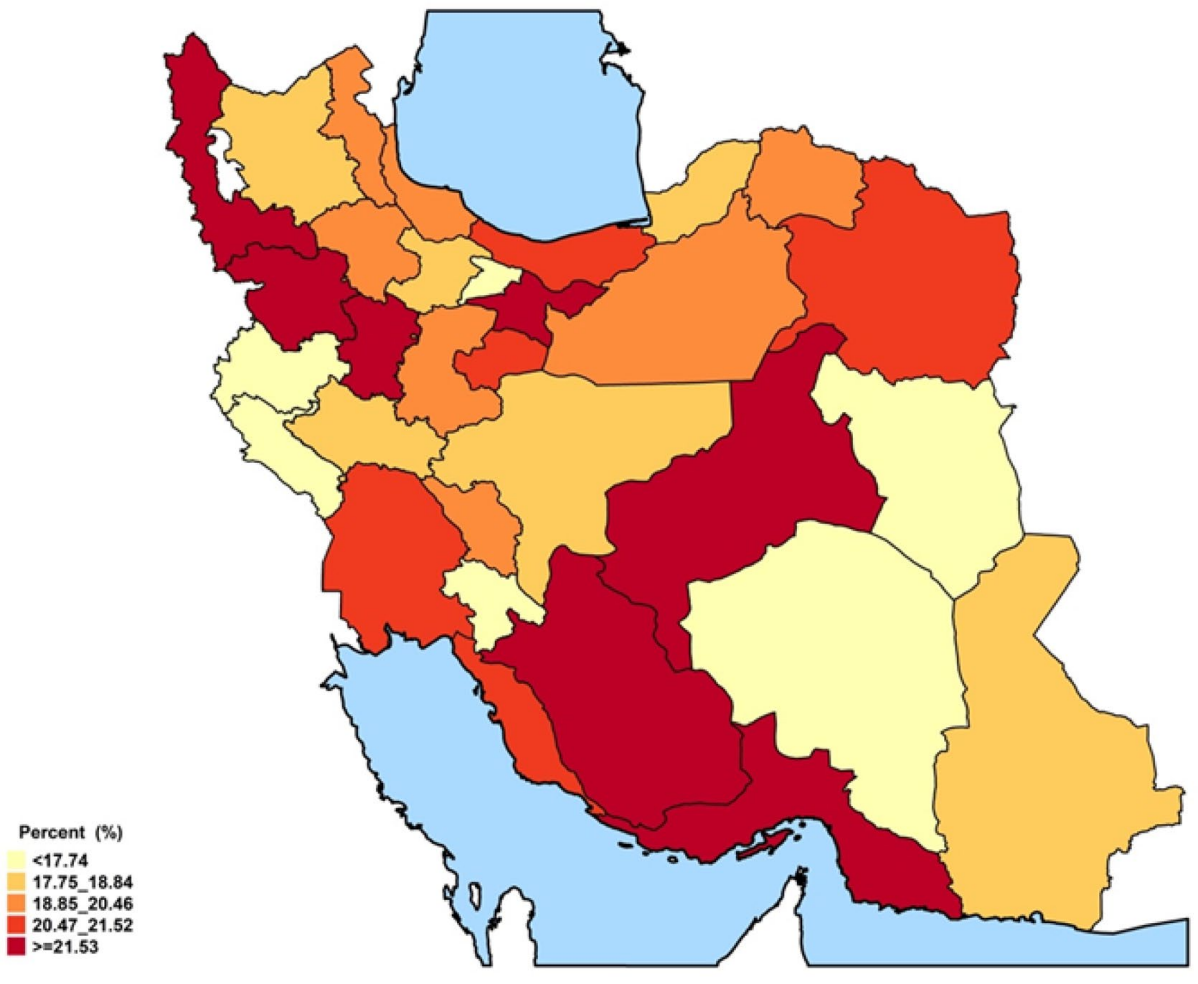
Prevalence of intermediate and high risk (10-year ASCVD risk ≥ 7.5%) population at sub-national and provincial levels. ASCVD: atherosclerotic cardiovascular disease. The figure was generated using ggplot2 from the R statistical package version 4.1.2 (https://cran.r-project.org).

Figure 5 depicts the provincial distribution of individuals with intermediate and high-risk ASCVD risk scores in Iran.

Substantial variations in the distribution of ASCVD risk were observed across Iranian provinces. The proportion of individuals at high risk (ASCVD ≥ 20%) ranged from 1.4% to 6.5%. Conversely, the low-risk category (ASCVD < 5%) varied from 61.5% to 77.2%. (Online Resource 1, Supplementary Table S1).

### National gaps in statin use and cardiometabolic treatment

In participants with a history of ASCVD, 56.5% were on statin therapy. (Table 1) However, in 40–75-year-old individuals with no ASCVD and no DM, only 9.9% and 5.1% of intermediate and high-risk groups for ASCVD were receiving statins, respectively. In subjects older than 40 years old, with DM and no ASCVD, statin intake was reported in 27.0%.

While hypertension was diagnosed in 77.0% of the population with ASCVD, 56.8% of participants with ASCVD were aware of hypertension, 52.9% reported receiving medication for it, and only 23% of total ASCVD individuals were considered to be in effective care. These numbers for the population with ASCVD and DM were 34.7% for prevalence, 29.2% for awareness, 26.4% for coverage, and 8.8% for receiving effective care, respectively. (Table 1).

In the population with no ASCVD, hypertension was diagnosed in 47.4% to 94.4% of the participants across different ASCVD risk groups, 16.7% to 54.81% of total participants in these groups were aware of hypertension, 13.1% to 47.2% reported receiving medication for it, and only 2.9% to 5.3% of total individuals in these groups were considered to be in effective care as defined by SBP < 130mmHg and DBP < 80mmHg. For DM, these numbers for the populations across different ASCVD risk groups were 7.2% to 59.3% for prevalence, 5.5% to 44.27% for awareness, 4.8% to 38.4% for coverage, and 2.5% to 16.8% for receiving effective care (HbA1c < 8), respectively. For hypercholesterolemia, the corresponding figures were 33.5% to 48.7% for prevalence, 15.2% to 27.5% for awareness, 9.9% to 22.6% for coverage, and 7.7% to 16.7% for effective care. (Table 3).

### Cardiovascular health metrics by ASCVD risk category

Regarding CVH metrics based on the LS7 criteria, among participants with estimated borderline and intermediate ASCVD risks, the most common number of ideal CVH metrics was 2, observed in 35.8% and 36.8% of these groups, respectively. This was 1 for the individuals with a high estimated ASCVD risk. (Table 4) According to the CVH metric criteria, poor blood pressure control was observed in 41.1% of subjects with borderline ASCVD risk, 54.6% with intermediate risk, and 76.4% with high ASCVD risk. Regarding FPG, 25.9% of participants with intermediate ASCVD risk and 49.7% of those with high ASCVD risk scored poorly (≥ 126). Across all ASCVD risk categories, intermediate BMI and physical activity scores had the highest prevalence, though nearly 30% of participants exhibited poor scores for both risk metrics. Although smoking was in an ideal state in borderline, intermediate, and high-risk ASCVD groups, 43.6% of individuals categorized as high risk for ASCVD still had a poor score. In the healthy diet, over 60% of subjects in all groups scored poorly. Participants were found to have the highest scores in smoking and cholesterol status, with more than 50% in the ideal group across all ASCVD risk categories.

**Table 4:** The Life Simple 7 metrics in participants with no report of atherosclerotic cardiovascular disease.

Variable		Age				
		20–39 years (N = 11,002)	40–75 years (N = 10,298)			
			ASCVD < 5% N = 6624 (64.34%) (63.3–65.36)	5% ≤ ASCVD < 7.5% N = 1154 (11.15%) (10.49–11.84)	7.5% ≤ ASCVD < 20% N = 1942 (19.1%) (18.26–19.97)	20% ≤ ASCVD N = 578 (5.42%) (4.95–5.92)
BMI (kg/m^2^)	≥ 30	1814 (16.6%) (15.84–17.4)	2148 (31.67%) (30.19–33.19)	333 (27.38%) (24.18–30.82)	529 (29.52%) (26.21–33.05)	150 (26.78%) (22.29–31.8)
	25- < 30	3652 (33.66%) (32.68–34.67)	2650 (41.51%) (39.87–43.18)	471 (41.77%) (37.9–45.74)	779 (39.89%) (36.56–43.31)	248 (43.52%) (38.05–49.16)
	< 25	5472 (49.73%) (48.68–50.78)	1820 (26.82%) (25.37–28.32)	349 (30.85%) (27.22–34.75)	631 (30.6%) (27.59–33.78)	180 (29.7%) (24.9–34.99)
Physical activity	All others	3082 (28.71%) (27.77–29.67)	1946 (32.04%) (30.48–33.64)	318 (31.02%) (27.29–35.01)	580 (30.82%) (27.63–34.21)	176 (32.32%) (27.33–37.75)
	1–149 min/week moderate intensity, 1–74 min/week vigorous intensity, 1–149 min/week moderate + vigorous	5933 (53.63%) (52.59–54.67)	3813 (56.31%) (54.65–57.95)	656 (55.48%) (51.48–59.41)	1109 (57.68%) (54.19–61.1)	337 (57.01%) (51.45–62.4)
	≥ 150 min/week moderate intensity, ≥ 75 min/week vigorous intensity, or ≥ 150 min/week moderate/vigorous	1961 (17.66%) (16.88–18.46)	855 (11.65%) (10.72–12.66)	179 (13.5%) (11.21–16.17)	247 (11.49%) (9.58–13.73)	65 (10.67%) (7.9–14.26)
Blood pressure (mmHg)	Systolic ≥ 140 or Diastolic ≥ 90	919 (7.91%) (7.37–8.49)	1514 (21.85%) (20.52–23.25)	515 (41.13%) (37.32–45.05)	1121 (54.63%) (51.12–58.09)	439 (76.45%) (71.51–80.76)
	Systolic: 120 ≤ < 140, Diastolic: 80 ≤ < 90, or treated to goal	4180 (38.32%) (37.31–39.35)	3095 (47.14%) (45.49–48.81)	502 (45.88%) (41.98–49.83)	700 (37.52%) (34.25–40.91)	131 (22.35%) (18.11–27.26)
	Systolic < 120/ Diastolic < 80 untreated	5855 (53.76%) (52.72–54.81)	2015 (31%) (29.5–32.54)	137 (12.99%) (10.32–16.23)	121 (7.85%) (5.8–10.54)	8 (1.2%) (0.54–2.64)
Fasting plasma glucose (mg/dL)	≥ 126	134 (2.5%) (1.98–3.15)	370 (6.47%) (5.6–7.46)	180 (15.99%) (13.29–19.11)	484 (25.94%) (23.14–28.95)	280 (49.73%) (44.19–55.29)
	100 ≤ < 126 or treated to goal	982 (18.59%) (17.11–20.16)	1974 (30.71%) (29.17–32.28)	389 (33.8%) (30.3–37.49)	623 (33.77%) (30.33–37.39)	136 (25.7%) (21.09–30.92)
	< 100 untreated	4630 (78.91%) (77.28–80.45)	4280 (62.83%) (61.18–64.45)	585 (50.21%) (46.26–54.16)	835 (40.29%) (37–43.68)	162 (24.56%) (19.85–29.97)
Total cholesterol (mg/dL)	≥ 240	122 (2.2%) (1.73–2.79)	308 (4.42%) (3.81–5.12)	74 (6.54%) (4.86–8.74)	136 (8.94%) (6.19–12.74)	56 (10.68%) (7.91–14.28)
	200 ≤ < 240 or treated to goal	650 (11.29%) (10.19–12.5)	1896 (29.1%) (27.61–30.64)	396 (32.61%) (29–36.43)	716 (36.34%) (33.23–39.56)	229 (38.05%) (32.96–43.43)
	< 200 untreated	4968 (86.51%) (85.21–87.71)	4419 (66.48%) (64.89–68.03)	684 (60.85%) (56.93–64.64)	1090 (54.72%) (51.18–58.22)	293 (51.26%) (45.72–56.78)
Smoking	Current smoking	1455 (14.14%) (13.41–14.89)	425 (6.22%) (5.5–7.03)	221 (18.38%) (15.52–21.64)	527 (26.47%) (23.73–29.42)	255 (43.66%) (38.12–49.37)
	Former, quit in the 12 months	578 (5.38%) (4.92–5.87)	300 (4.41%) (3.78–5.13)	73 (5.42%) (4.15–7.05)	135 (6.88%) (5.39–8.74)	26 (4.59%) (2.92–7.15)
	Never or quit in more than 12 months ago	8951 (80.48%) (79.64–81.31)	5899 (89.37%) (88.34–90.33)	860 (76.2%) (72.78–79.3)	1280 (66.65%) (63.46–69.69)	297 (51.74%) (46.15–57.3)
Healthy diet score	0–1	3753 (68.09%) (66.43–69.69)	4278 (65.56%) (63.98–67.11)	749 (63.19%) (59.22–66.99)	1244 (65%) (61.71–68.15)	389 (68.66%) (63.32–73.55)
	2–3	1914 (31.49%) (29.89–33.13)	2270 (34%) (32.46–35.58)	390 (36.44%) (32.65–40.4)	672 (34.45%) (31.31–37.73)	177 (30.72%) (25.86–36.04)
	4–5	24 (0.43%) (0.25–0.73)	31 (0.44%) (0.28–0.69)	4 (0.38%) (0.11–1.24)	10 (0.56%) (0.27–1.15)	3 (0.63%) (0.17–2.32)
Number of ideal CVH metrics	0	245 (0.53%) (0.34–0.82)	26 (0.38%) (0.23–0.63)	13 (1.08%) (0.54–2.13)	76 (3.76%) (2.85–4.95)	52 (8.93%) (6.58–12.01)
	1	1365 (3.56%) (2.96–4.28)	683 (10.47%) (9.52–11.5)	221 (17.86%) (15.23–20.84)	470 (25.44%) (22.3–28.86)	203 (36.21%) (31.15–41.6)
	2	2586 (13.26%) (11.91–14.74)	1813 (27.92%) (26.42–29.48)	395 (35.83%) (32.1–39.74)	693 (36.81%) (33.55–40.2)	191 (34.82%) (29.32–40.75)
	3	3349 (29.24%) (27.47–31.07)	2100 (32.65%) (31.09–34.25)	337 (27.73%) (24.44–31.28)	485 (24.05%) (21.25–27.09)	105 (16.25%) (12.91–20.25)
	4	2154 (32.01%) (30.35–33.71)	1363 (19.45%) (18.24–20.73)	164 (15.29%) (12.38–18.74)	187 (8.83%) (7.05–11.01)	27 (3.79%) (2.49–5.73)
	5	1157 (19.18%) (17.84–20.59)	576 (8.31%) (7.48–9.22)	24 (2.21%) (1.22–3.99)	31 (1.11%) (0.72–1.69)	0
	6	146 (2.23%) (1.82–2.73)	63 (0.82%) (0.6–1.11)	0	0	0

Data are presented as mean (confidence interval) or for continuous variables and n (% and confidence interval) for categorical variables as appropriate.

Healthy diet score components: (1) fruits and vegetables, ≥ 31.5 cups/week, (2) fish ≥ 1–3 times/week, (3) whole grains ≥ 1–2 times/day, (4) sodium intake, ≤ 1.5 g/d, and (5) sugar-sweetened beverages ≤ 1–3 times/week.

ASCVD, Atherosclerotic cardiovascular diseases; BMI, Body mass index; CVH, cardiovascular health metric.

## Discussion

In this study with a nationally representative population in Iran, we found that 7.4% of the 27,822 participants had ASCVD, corresponding to approximately 4.32 million individuals nationally. Also, among the remaining 40–75-year-old participants without ASCVD, 11.1% had a borderline risk of developing ASCVD over the next 10 years, 19.1% had an intermediate risk, and 5.4% had a high risk. Metabolic risk factors were high among both participants with ASCVD and subjects at a high risk of developing one. Additionally, the results revealed significant gaps in ASCVD risk management, with suboptimal statin use, alongside poor blood pressure, DM control, and lifestyle metric scores across all risk groups.

The NAME region ranked second globally in IHD burden in 2019^9,26^. Accordingly, Iran had a high age-standardized prevalence and incidence rate of IHD in the NAME region in 2021^8^. The overall 7.4% ASCVD rate in our study is higher than the 5.0% rate of self-reported heart attack or chest pain from heart disease, or stroke in individuals aged ≥ 15 years reported by the 2017 STEPS study in Turkey^27^. Another study using National Health and Nutrition Examination Survey (NHANES) 2015–2018 data on individuals ≥ 20 years in the United States reported an ASCVD prevalence of 7.5^28^. Iran’s elevated ASCVD prevalence, surpassing Turkey and aligning closely with the United States, reflects a significant cardiovascular health challenge shaped by a complex mix of risk factors and systemic influences. While these national figures highlight the scale of the issue, variations in prevalence and contributing factors across provinces may reveal significant opportunities for targeted public health efforts.

At the provincial level, substantial differences were observed in the ASCVD prevalence and recent annual event rate in Iran, reflecting underlying inequalities in risk factors and access to care. Previous studies have documented marked variations in the prevalence of hypertension and DM, as well as differences in treatment coverage and effectiveness across the country^22,29,30^. Limited access to cardiovascular care, a challenge particularly acute in developing nations like Iran, further exacerbates these disparities^31^. Socioeconomic factors, including geography, ethnicity, income, and education, also play a critical role, as evidenced by research in both high-income and developing countries^32,33^. These provincial differences underscore the need for targeted interventions to address the uneven burden of ASCVD in Iran. Accordingly, prevention policies should be tailored to each province’s specific gaps and align with calls to address cardiometabolic drivers, including obesity and diabetes, through organized prevention programs^34,35^.

Assessing and predicting future CVD risk can serve as another principal component of primary prevention strategies. Previous studies have examined the trends in CVDs and their projections in Iran. In a study conducted on employees of a university aged 25–70 years in the central part of Iran in 2021, the intermediate- and high-risk estimated 10-year ASCVD rates were lower (13.2% vs. 19.1% for intermediate risk and 2.5% vs. 5.4% for high-risk, respectively for the previous and current study). This difference may have been due to the probable role of education^36^. In another report, the CVD risk predictions were evaluated using World Health Organization (WHO) risk scores derived from Iran’s STEPS data. In 2016, 31.5% of men and 26.2% of women were considered high-risk^37^. Another study using the STEPS surveys from 2005 to 2016 revealed that in 2016, nearly 15.0% of the population aged 30–74 had a significant (> 20%) risk of developing CVD, according to the Framingham model^18^. In agreement with the current study, urban areas had a greater risk of ASCVD than rural regions^18^, demonstrating the probable role of physical inactivity^38,39^.

While both the AHA/ACC ASCVD and Framingham risk estimators calculate risk scores, their translational meanings and clinical implications can differ. This makes a direct comparison challenging. Nonetheless, the results of the previous studies and the current report highlight an alarming rate of 14% (> 20% according to Framingham risk score) and 24.5% (both intermediate and high risks according to the AHA/ACC ASCVD estimates) for the population aged 30–75 and 40–75 years old with seemingly no CVD across the whole country.

The current study’s ASCVD risk estimates in Iran were lower than those in the United States and Korea. A study conducted using NHANES data indicated that in 2018, participants aged 40–79 with intermediate and high age-standardized 10-year ASCVD risk comprised nearly 33% and 19% of the study population in the USA, respectively^40^. In another study using the NHANES 1999–2016, intermediate and high ASCVD risk populations were reported at 25.0% and 8.4%, respectively^41^. Evaluating the Korean National Health and Nutrition Examination Survey (KNHANES) 2009–2010 showed that in the population aged 40–79 years, 31.3% had an intermediate or high risk of developing ASCVD in the next ten years, using PCE scores^42^.

The distribution of high ASCVD risk scores across provinces differed from that observed in the study using Framingham scores^18^. However, similar in both studies, cities in the southeast and certain western provinces had a relatively lower risk. Although this was not explored in the current study, it may be due to various factors, such as better lifestyles and dietary habits in these areas. A concerning finding of our study was the high ASCVD recent annual event rate and estimated risks in the northwestern provinces, which currently have low established ASCVD prevalence. Notably, national studies have indicated high hypertension rates, dyslipidemia, DM, and low DM coverage in these districts^22,43,44^.

An interesting finding of the current study was that the geographical distribution of provinces with a high and low risk for ASCVD almost mirrored and corresponded to the geographical distribution of obesity and overweight rates, with the south and southeastern provinces being low risk and high rates of BMI < 25, and the northeast and central parts being high risk and having a high prevalence of obesity^43^. Although the Southeast and South provinces had low ASCVD prevalence, their recent annual event rate was rather high. This could be a result of high rates of DM, prediabetes, and hypertension across both males and females, and the high dyslipidemia among females in these areas, as reported by the national-level studies on these comorbidities^22,24,44^.

Regarding metabolic and behavioral derangements, participants with ASCVD had higher rates of DM, BMI, hypertension, and hyperlipidemia and lower rates of physical activity. Additionally, participants with a borderline, intermediate, and high risk of ASCVD also had high rates of these disorders. The increasing trend in the prevalence of obesity and BMI has been marked in previous national studies^43,45^. For other risk factors, DM and prediabetes have been shown to have a 39% prevalence in Iran, with only 65% treatment coverage. Moreover, among patients with previously diagnosed DM, 58.4% had fair glycemic control (HbA1c < 8%)^22,24^. For comparison, in the United States, the rate of linkage to diabetes care was 94% according to NHANES 2013–2016 data^46^, and fair glycemic control was achieved in 70.4% of the adult population with DM according to NHANES 2015–2018 data^47^. In Iran, the coverage and control rate of hypertension as a leading attributable cause of IHDs was 26% and 18%, according to the ACC/AHA guidelines in the STEPS 2021 study^44^. These numbers are lower than those reported from Turkey. According to the STEPS 2017 study in Turkey, 59% of the population with hypertension were aware of their disease, 46.2% were under treatment, and 23.9% were controlled^27,48^.

Aligning with previous studies, the current report revealed unsatisfactory treatment coverage rates of DM and hypertension in individuals with either ASCVD or at risk of developing ASCVD. In fact, the data exhibited a substantial gap between prevalence and awareness (e.g., 77.0% vs. 56.8% for hypertension in ASCVD; 34.7% vs. 29.2% for diabetes), with smaller gaps between awareness and treatment, but effective care lags significantly (23.0% and 8.8%, respectively) in participants with ASCVD. The notable gap in effective care for hypertension and diabetes in Iran suggests opportunities for improvement. This is especially important when compared with studies in high-resource settings, such as a report using NHANES 2015–2018 data, which reported that 76.9% of ASCVD patients had blood pressure < 140/90 mmHg and 84.0% had HbA1c < 7%^28^. The same finding was also revealed among individuals with high ASCVD risk. Notably, wide disparities exist between disease prevalence and patient awareness, with risk-dependent increases in these metrics yet continued shortfalls in effective control. Despite this, awareness, coverage, and effective care increased with higher ASCVD risk scores, indicating that preventive strategies are on the right track and reaching higher-risk individuals more effectively.

These worrying rates, both in the general and high-risk populations, necessitate the adoption of primary preventive measures to mitigate these amenable risk factors. To address gaps in hypertension and diabetes management among ASCVD and at-risk groups, policymakers should launch targeted community-based screening in high-burden areas to bridge the prevalence-awareness gap, improving early detection. Additionally, implementing quality metrics and digital tools will narrow the gap between treatment and effective care.

Our appraisal showed that individuals with ASCVD showed better dietary habits than those without, though both groups had suboptimal diets. Similar to the current study, the diet score has consistently ranked lowest among the other CVH parameters globally^49^. Suboptimal diets, driven by low fruit and vegetable intake and high sodium consumption, were a major cause of cardiometabolic mortality in 2011, affecting nearly 87% of Iranians who consumed less than the WHO-recommended amounts^50,51^. In a 2016 study, it was revealed that over 95% of Iranians consumed more than the WHO-recommended maximum of 5 g of salt per day by and 2.3 g per day as advised by the AHA^52^. Various strategies, including nutritional traffic light labeling, have been implemented to reduce harmful dietary factors^53,54^. However, tailored methods are still needed to improve heart-healthy dietary patterns^55^, including increasing the accessibility of favorable nutritional factors for lower-income families and raising awareness.

Various studies have illustrated the positive impacts of improving cardiovascular health metrics^56^. Similar to previous studies, the intermediate CVH (2–5 ideal metrics) was the most common category in the current report^49^. While a previous study using STEPS data (2007–2016) reported improvements in smoking, hypertension, and hypercholesterolemia trends^57^, our findings highlight ongoing concerns. Current smoking rates in the intermediate-risk and high-risk groups were respectively nearly two-fold higher, respectively, than the 13.7% rate reported in 2016. Additionally, nearly 50% of intermediate- or high-risk individuals had poor or intermediate cholesterol status, versus 20.4% in the 2016 study. Hypertension control was similarly concerning, with over 90% of intermediate-risk and 98% of high-risk individuals showing poor or intermediate hypertension control, compared with ~ 70% in the 2016 data.

Besides evaluating the primary modifiable risk factors and their management status, we also assessed the adoption of the 2019 guidelines on primary and secondary prevention of CVDs with statin therapy^19^. We found that only nearly 50% and 30% of the study population with ASCVD were receiving statins and aspirin, respectively. This was 27% and below 10% for patients with DM and participants with a high risk of developing ASCVD in the future, respectively. In a previous national study in Iran, the statin use among patients with DM was reported as 50%, close to the 60% global WHO target^22^. In the United States, data from more than 300,000 patients with ASCVD showed that 76.1% were receiving statins, a higher rate than the 50% observed in the current study^58^. Evaluating the data of nearly 70 million eligible individuals from the NHANES 2017–2020 for statin therapy as a primary prevention measure, Chobufo et al. showed that only 25.4% of individuals with intermediate ASCVD risk, 40.6% of subjects with high ASCVD risk, 45.2% of patients with DM, and 58.5% of the population with diagnosed ASCVD were receiving statins^59^. In comparison, in the current report, a lower percentage of the population in each category reported statin use. It should be noted that statin treatment thresholds and approaches vary across different guidelines. While lower eligibility rates in the 2022 USPSTF recommendation compared to the 2018 AHA/ACC guideline have been shown^60^, future research could explore how these guideline differences influence statin coverage in Iran^61^. These data highlight the need to raise awareness of possible undertreatment and the potential to improve current prescribing practices for statins and aspirin for clinicians and policymakers.

From a policy and strategy standpoint, previous studies have proposed strategies to tackle cardiovascular issues in Iran. The establishment of population-based strategies within high-risk populations has been proposed as an answer to this question. This approach leverages support from both approaches without widening health inequalities by focusing on a specific population^18,30,62^. However, national studies evaluating the population attributable fraction (PAF) of CVDs across different levels of FPG, blood pressure, and plasma total cholesterol have shown that the CVD PAF estimated for pre-high-risk individuals in these parameters did not differ significantly from that for high-risk individuals. These important findings underscore the need to extend preventive strategies to pre-high-risk groups to mitigate ASCVD risk across all risk strata in Iran^63–65^.

Iran’s 2014 health reform and strategies, including cost-effective health packages at the primary level^66^ and successful measures to control IHDs at the secondary and tertiary levels^7^, are among the efforts to address the ASCVD burden, either directly or indirectly. Recently, the first national clinical guideline for acute coronary syndrome was developed^67^. The Iran-package of essential non-communicable disease (IraPEN) program has also shown modest reductions in CVD risk scores^68^. Trials on the implementation of polypill use, like PolyIran, have also demonstrated a significant reduction in CVD events^69^. Food Traffic Light Labeling, mandated from 2016, targeted unhealthy diet habits as another solution to tackle cardiometabolic risk factors^70^.

The management of ASCVD at the primary level should be responsive to the specific structure and features of each country^71^. However, successful national plans from other countries could provide additional insights for adaptation in Iran: Finland’s North Karelia Project achieved major declines in CVD mortality through population-based measures to address several modifiable risk factors^72^. Japan’s Specific Health Checkups and Specific Health Guidance are among other successful strategies^73^. Other strategies, such as front-of-pack labeling, mandatory food reformulation, and tobacco tax increases, have proven effective globally for controlling risk factors^71^. The Singapore Healthier SG program and integration of hospitals with primary care services are other examples of successful plans^74^.

Overall, remarkable strides have been made in tackling NCDs in Iran through various effective public health-driven strategies^4,75^. However, the demographic and epidemiologic transition of the country contributes to the unmet need to alleviate the modifiable factors that remain^5^. The emerging pandemic of NCDs and concerning rates of present and future ASCVD call for multi-sectoral collaborations, resource-driven reinforcement of health infrastructures, and sustainable population-based interventions that need to be integrated into national health endeavors.

### Limitations

The present study has some limitations despite its large scale. First, there has been debate on the efficacy and performance of various ASCVD risk scores and their clinical applications^76^. However, the added benefits and clinical applicability of the 2013 ACC/AHA risk models have been proven in large studies^77,78^. Although they are at risk of overestimation in certain populations^79^, including those using statins and aspirin, the risk was relatively low in the current study. Additionally, the health-conscious nature of the research population has been proposed as another reason. This might not be the case since the research population in this study was referred to. Nonetheless, because potential overestimation may persist across populations, our estimates should be interpreted primarily as population-level risk stratification rather than as precise absolute event probabilities. This is particularly relevant in the context of the global rise in metabolically driven CVD, which can change baseline risk and model calibration across settings^80,81^. This should be considered when interpreting the observed risk scores for clinical decision-making and when generalizing these estimates to other populations. The various definitions of CVD and ASCVD used across studies made direct comparison difficult. However, by adopting a standard questionnaire format, we reduced this limitation as much as possible. The use of self-reported dietary data obtained through interviewer-administered questionnaires was another limitation. It may be subject to recall inaccuracies, social desirability influences, and inherent challenges in estimating sodium intake from behavioral questions, which may potentially affect the classification of dietary components within the adapted LS7 framework. Furthermore, self-reported data on ASCVD prevalence and risk factors may be prone to under- or over-reporting, with potential for differential misclassification across subgroups such as those varying in education or health literacy. To minimize such influences, we employed standardized questionnaires developed through rigorous methods and tested globally, and provided staff training. Finally, data collection overlapped with the COVID-19 period, during which patterns of healthcare use and everyday behaviors likely differed from typical years^82^. These findings should therefore be interpreted in light of potential Pandemic-era changes, including fewer routine visits, shifts in physical activity and diet, and episodic alterations in chronic-care follow-up, which may have affected risk-factor control and preventive management^83^.

## Conclusion

In conclusion, Iran has a relatively high ASCVD prevalence, with the potential to increase in the coming years based on ASCVD risk estimates. The demonstrated correlation between high-risk cities and known amenable risk factors made them a good target for future preventive strategies. The drastic rates of NCDs and inadequate primary and secondary preventive measures, combined with low adherence to AHA/ACC guidelines in the management of participants with either ASCVD or at high risk, were demonstrated. Finally, the urgent need to introduce tailored health strategies was emphasized.

## Supplementary Information

Below is the link to the electronic supplementary material.

Supplementary Material 1

## Data Availability

The datasets created and/or evaluated in this study are not publicly accessible. However, they may be obtained from the corresponding author upon reasonable request. All data inquiries should be directed to the corresponding author.
