# Correction: Epidemiology and cardiometabolic care in adults with ASCVD and high 10-year ASCVD risk: 2021 WHO STEPS study in Iran

**DOI:** 10.1038/s41598-026-50915-2

**Published:** 2026-04-29

**Authors:** Hossein Farrokhpour, Maryam Nasserinejad, Naser Ahmadi, Ali Golestani, Mohammad Keykhaei, Sina Azadnajafabad, Arya Aminorroaya, Mohammadreza Naderian, Nazila Rezaei, Shirin Djalalinia, Yosef Farzi, Mohammad-Mahdi Rashidi, Negar Rezaei, Erfan Ghasemi, Elham Abdolhamidi, Moein Yoosefi, Ameneh Kazemi, Rosa Haghshenas, Mohamad Taghi Majnoon, Farshad Farzadfar

**Affiliations:** 1 Non-communicable Diseases Research Center, Endocrinology and Metabolism Population Sciences Institute, Tehran University of Medical Sciences, Tehran, Iran; 2 Development of Research and Technology Center, Deputy of Research and Technology, Ministry of Health and Medical Education, Tehran, Iran; 3 Endocrinology and Metabolism Research Center, Endocrinology and Metabolism Clinical Sciences Institute, Tehran University of Medical Sciences, Tehran, Iran; 4 Division of Pediatric Cardiology, Pediatrics Center of Excellence, School of Medicine, Children’s Medical Center, Tehran University of Medical Sciences, Tehran, Iran

Correction to: *Scientific Reports* 10.1038/s41598-026-45344-0, published online 27 March 2026

The original version of this Article contained an error in the name of author Sina Azadnajafabad which was incorrectly given as Sina Azadnajadabad.

The original Article has been corrected.

