# Supplementary material for: Epidemiology and cardiometabolic care in adults with ASCVD and high 10-year ASCVD risk: 2021 WHO STEPS study in Iran
Source: Sci Rep. 2026 Mar 27;16:10825. doi: 10.1038/s41598-026-45344-0 (PMC13039966; doi:10.1038/s41598-026-45344-0)
Supplement: Supplementary file 1 — Supplementary Material 1 [file 41598_2026_45344_MOESM1_ESM.pdf]

Epidemiology and Cardiometabolic Care in Adults with ASCVD and High 10-Year ASCVD Risk: 2021 WHO STEPS Study in Iran

Online Resource 1 - Supplementary Materials

Table of Contents	Page No.
Supplementary Method Information	2
Supplementary Figure S1	3
Supplementary Figure S2	4
Supplementary Figure S3	5
Supplementary Figure S4	6
Supplementary Table S1	7-8

Supplementary Method Information:

For the healthy diet component of Life's Simple 7 (LS7), scoring was adapted to the STEPS survey and categorized into poor, intermediate, and ideal levels. A minor adjustment was made by replacing whole grains with fiber-rich whole grains to match the corresponding item in the STEPS questionnaire. The healthy diet score included five components, with one point awarded for meeting each one: fruits and vegetables (ideal: ≥31.5 cups/week), fish (ideal: ≥1–3 times/week), whole grains (ideal: ≥1–2 times/day), sodium intake (ideal: ≤1.5 g/day), and sugar-sweetened beverages (ideal: ≤1–3 times/week). The five binary scores were summed to produce a total healthy diet score ranging from 0 to 5, which was then classified as poor (0–1), intermediate (2–3), or ideal (4–5). Data for these components were extracted from STEPS diet/nutrition items as described in the following: the STEPS survey asked about the daily number of main meals and snacks. Breakfast consumption was assessed as days per week using the options "Always", "5 to 6 days", "3 to 4 days", "1 to 2 days", and "Never". For fruit and vegetable intake, participants reported the number of days per week they consumed fruits and vegetables and the number of servings they typically had on those days. One standard serving was considered as 80 grams and was converted to cup-equivalents using the type of produce and standard cup sizes commonly used in Iran. The mean daily servings of fruits and vegetables were calculated as (days per week consuming fruits or vegetables) × (servings consumed on a typical day) / 7. Usual intake frequency for fish, whole grains (including pasta, brown rice, barley bread, and grain bread), red meat (such as goat, sheep, camel, beef, veal, and lamb), sugar-sweetened beverages (such as industrial juices, soft drinks, and energy drinks), processed meat (such as kielbasa and sausage), dairy products (including cheese, milk, and yogurt), nuts/seeds, rice, and bread was collected using a questionnaire with the following response options: "more than 3 times a day", "1 or 2 times a day", "4 to 6 times a week", "1 to 3 times a week", "1 to 3 times a month" and "less than 1 time a month or never". Participants also reported the type of dairy they most often consumed, selecting from "High fat", "Low fat", "2.5% fat", and "Native dairy".

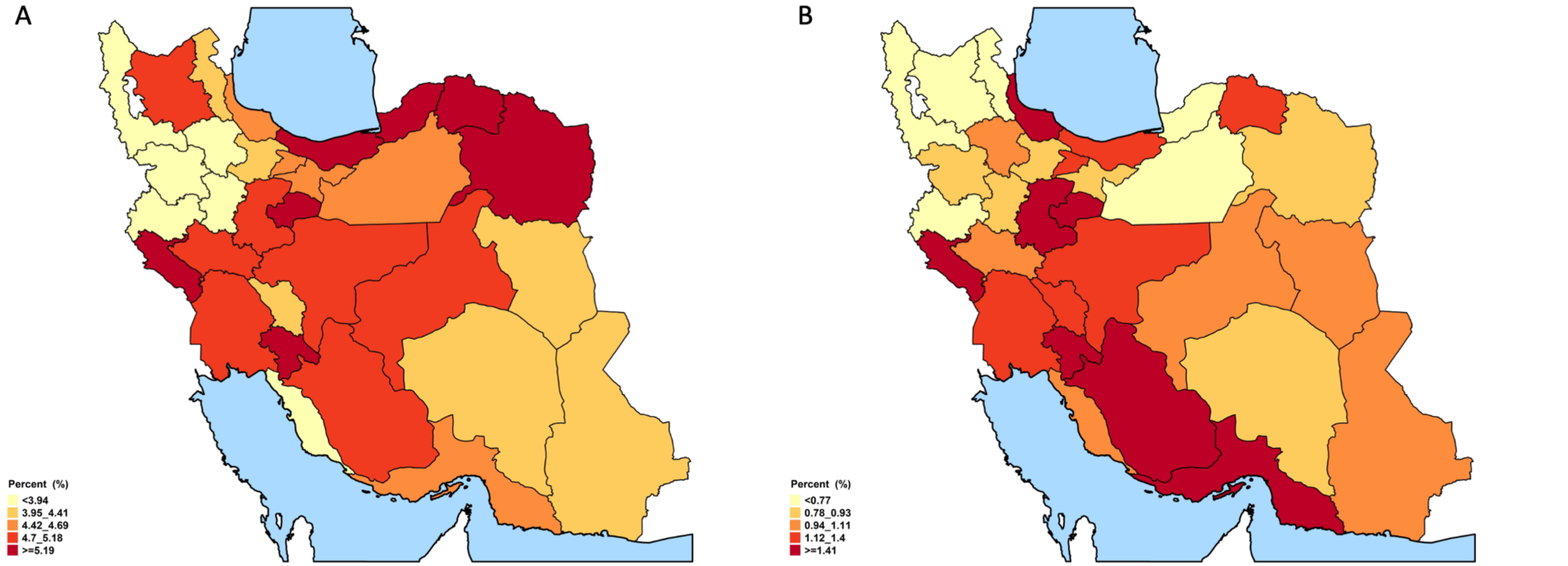

Supplementary Figure S1: Subnational and provincial prevalence of coronary artery diseases (A), stroke (B).

The figures were generated using ggplot2 from the R statistical package version 4.1.2 (https://cran.r-project.org).

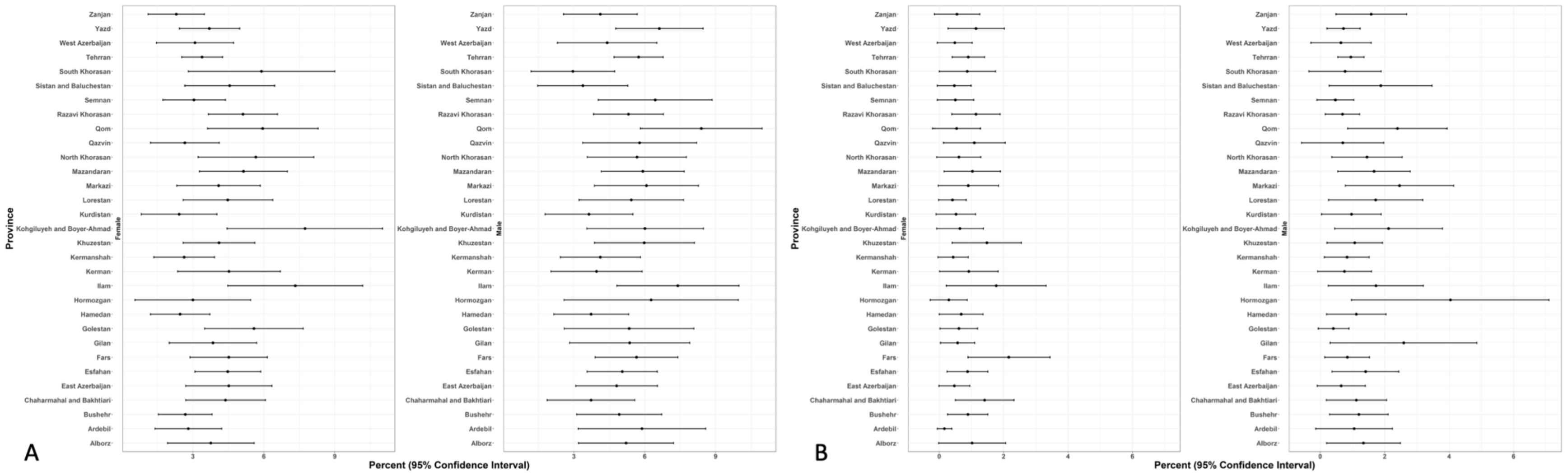

Supplementary Figure S2: Gender and geographical distribution of prevalence of coronary artery diseases (A), stroke (B)

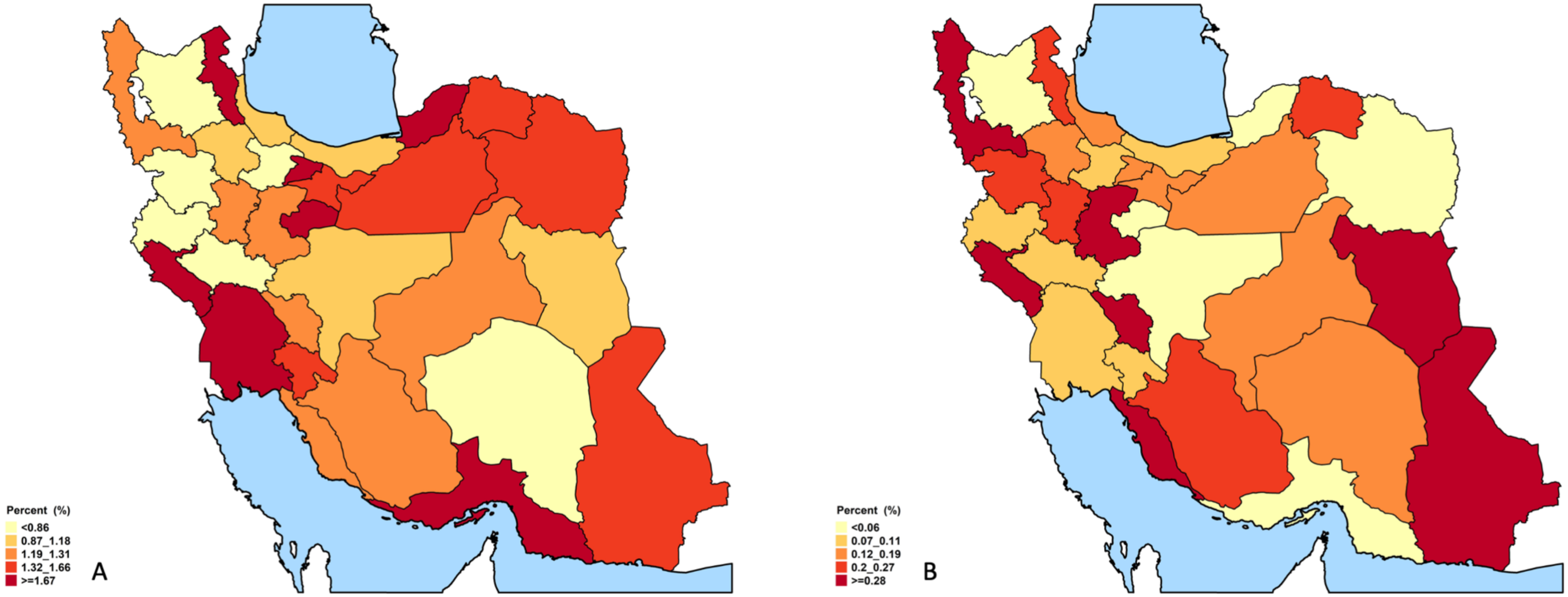

Supplementary Figure S3: Subnational and provincial recent annual event rate of coronary artery diseases (A), stroke (B).

The figures were generated using ggplot2 from the R statistical package version 4.1.2 (https://cran.r-project.org).

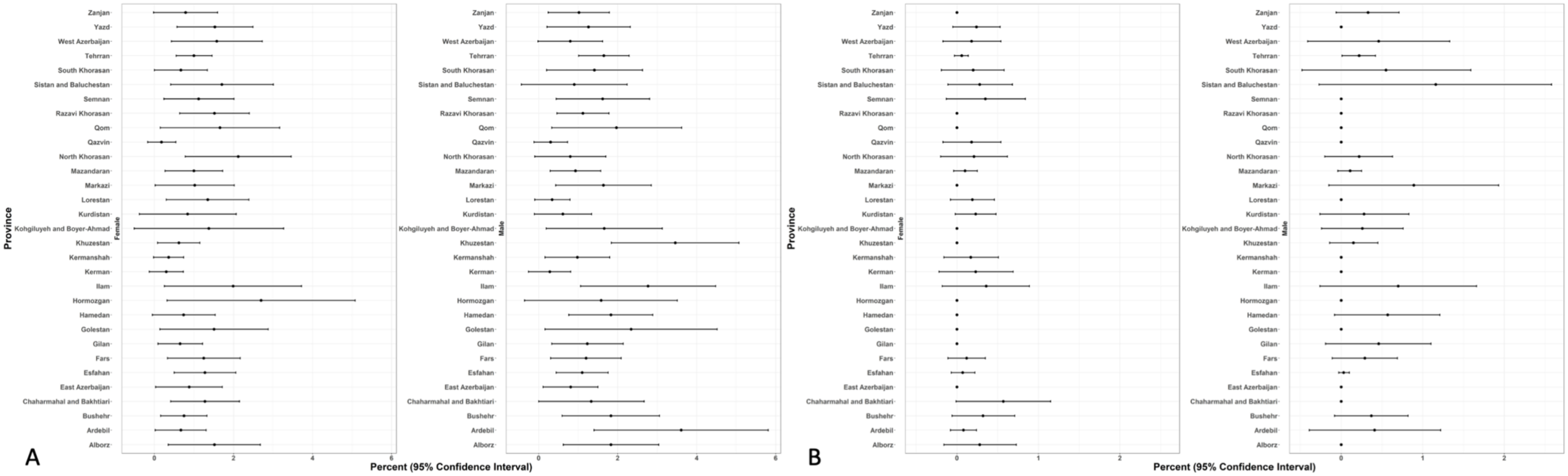

Supplementary Figure S4: Gender and geographical distribution of recent annual event rate of coronary artery diseases (A), stroke (B)

Supplementary Table S1. The prevalence of ASCVD risk scores among participants aged 40-75 years with no reported ASCVD according to different provinces.

**Province**	**ASCVD <5%**	**5% ≤ ASCVD <7.5%**	**7.5% ≤ ASCVD <20%**	**ASCVD ≥20%**
Markazi	74.87% (70.26-78.98)	8.35% (5.64-12.2)	14.26% (10.76-18.66)	2.52% (1.47-4.28)
Gilan	70.11% (63.87-75.68)	9.93% (6.59-14.7)	16.3% (11.55-22.5)	3.66% (2.15-6.17)
Mazandaran	66.58% (61.1-71.64)	14.65% (10.7-19.74)	15.43% (11.68-20.12)	3.34% (1.94-5.69)
East Azerbaijan	72.6% (66.87-77.66)	11.53% (7.2-17.97)	11.47% (8.51-15.28)	4.4% (2.87-6.7)
West Azerbaijan	69.15% (64.31-73.61)	9.88% (6.72-14.31)	16.8% (13.41-20.85)	4.16% (2.68-6.39)
Kermanshah	73.72% (68.48-78.36)	12.71% (8.65-18.29)	11.64% (8.47-15.78)	1.94% (0.91-4.09)
Khuzestan	67.4% (62.97-71.55)	12.2% (8.5-17.22)	16.86% (13.67-20.61)	3.53% (2.14-5.79)
Fars	70.25% (66.22-73.99)	10.23% (7.65-13.55)	17.16% (14.18-20.61)	2.36% (1.42-3.89)
Kerman	67.04% (62.11-71.62)	13.65% (9.72-18.85)	16.75% (12.86-21.52)	2.56% (1.34-4.84)
Razavi Khorasan	68.63% (64.6-72.39)	12.81% (9.75-16.65)	15.71% (12.96-18.91)	2.86% (1.83-4.44)
Esfahan	72.11% (68.85-75.15)	9.91% (7.63-12.77)	14.66% (12.37-17.3)	3.32% (2.34-4.69)
Sistan and Baluchestan	70.75% (65.76-75.29)	11.85% (8.2-16.83)	14.08% (10.52-18.58)	3.32% (2.02-5.42)
Kurdistan	61.5% (55.48-67.19)	13.2% (8.63-19.68)	21.04% (16.75-26.09)	4.25% (2.63-6.81)
Hamedan	71.23% (66.3-75.7)	7.89% (5.1-12.01)	15.12% (11.65-19.41)	5.76% (3.61-9.08)
Chaharmahal and Bakhtiari	72.2% (68.09-75.97)	8.58% (5.91-12.3)	15.92% (12.96-19.41)	3.3% (2.13-5.07)
Lorestan	69.65% (64.96-73.96)	11.7% (8.44-15.99)	14.28% (10.9-18.48)	4.38% (2.63-7.22)
Ilam	71.49% (65.12-77.1)	11.91% (8.07-17.23)	14.69% (10.21-20.69)	1.91% (0.89-4.06)
Kohgiluyeh and Boyer-Ahmad	70.53% (65.24-75.32)	13.98% (10.06-19.1)	11.12% (7.84-15.54)	4.38% (2.54-7.43)
Bushehr	67.15% (61.97-71.94)	13.27% (9.57-18.11)	14.37% (10.97-18.6)	5.22% (3.4-7.91)
Zanjan	69.48% (65.56-73.14)	11.71% (9.03-15.05)	14.73% (11.94-18.03)	4.08% (2.72-6.09)
Semnan	70.59% (64.73-75.84)	11.4% (7.64-16.67)	14.94% (10.73-20.42)	3.07% (1.86-5.01)

Yazd	70.96% (63.98-77.08)	7.71% (4.55-12.79)	16.52% (11.86-22.55)	4.8% (2.92-7.81)
Hormozgan	65.19% (58.85-71.04)	10.86% (7-16.49)	17.42% (12.43-23.86)	6.53% (3.95-10.61)
Tehran	70.71% (66.51-74.58)	8.9% (6.72-11.69)	17.7% (14.25-21.77)	2.7% (1.56-4.62)
Ardebil	71.81% (67.08-76.11)	11.03% (7.99-15.03)	12.48% (9.14-16.8)	4.68% (2.84-7.62)
Qom	77.27% (71.55-82.12)	5.95% (3.61-9.64)	13.38% (9.49-18.53)	3.4% (1.61-7.05)
Qazvin	70.12% (64.83-74.92)	13.72% (9.93-18.65)	13.9% (10.42-18.31)	2.26% (1.14-4.46)
Golestan	71.35% (67.56-74.86)	11.18% (8.59-14.42)	13.68% (10.92-16.99)	3.79% (2.5-5.71)
North Khorasan	71.89% (67.44-75.94)	12.42% (9.33-16.34)	13.13% (10.08-16.92)	2.57% (1.44-4.56)
South Khorasan	73.09% (68.14-77.52)	9.2% (6.4-13.07)	14.5% (10.96-18.92)	3.21% (1.91-5.34)
Alborz	75.37% (68.36-81.25)	12.69% (8.14-19.25)	10.52% (6.77-16)	1.42% (0.63-3.17)

Data are presented as percentages (confidence interval).

ASCVD: atherosclerotic cardiovascular disease.
